# Sialosides Containing 7-*N*-Acetyl Sialic Acid Are Selective Substrates for Neuraminidases from Influenza A Viruses

**DOI:** 10.1021/acsinfecdis.2c00502

**Published:** 2022-12-01

**Authors:** Anoopjit Singh Kooner, Yue Yuan, Hai Yu, Hyeog Kang, Laura Klenow, Robert Daniels, Xi Chen

**Affiliations:** † Department of Chemistry, University of California, One Shields Avenue, Davis, California 95616, United States; ‡ Division of Viral Products, Center for Biologics Evaluation and Research, Food and Drug Administration, Silver Spring, Maryland 20993, United States

**Keywords:** carbohydrate, chemoenzymatic synthesis, *N*-acetyl analogue, *O*-acetyl sialic acid, sialidase, influenza A virus neuraminidase

## Abstract

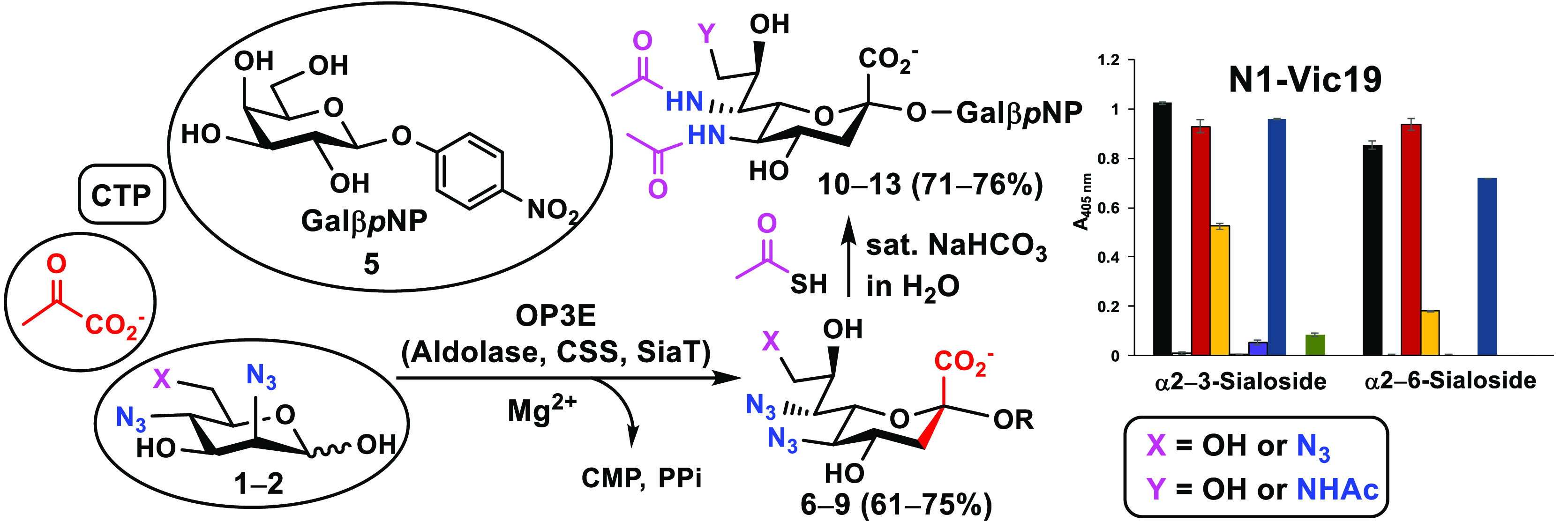

Sialidases or neuraminidases are sialic-acid-cleaving enzymes that are expressed by a broad spectrum of organisms, including pathogens. In nature, sialic acids are monosaccharides with diverse structural variations, but the lack of novel probes has made it difficult to determine how sialic acid modifications impact the recognition by sialidases. Here, we used a chemoenzymatic synthon strategy to generate a set of α2–3- and α2–6-linked sialoside probes that contain 7-*N*-acetyl or 7,9-di-*N*-acetyl sialic acid as structure mimics for those containing the less stable naturally occurring 7-*O*-acetyl- or 7,9-di-*O*-acetyl modifications. These probes were used to compare the substrate specificity of several sialidases from different origins. Our results show that 7-*N*-acetyl sialic acid was readily cleaved by neuraminidases from H1N1 and H3N2 influenza A viruses, but not by sialidases of human or bacterial origin, thereby indicating that the influenza enzymes possess a distinctive and more promiscuous substrate binding pocket.

Sialic acids (Sias) are nine-carbon α-keto acids that are part of the nonulosonic acid (NulO) family.^1^ In nature, Sias display significant structural diversity and are commonly found as terminal monosaccharides on glycan components of glycoproteins and glycolipids from vertebrates and higher invertebrates. They are also part of the repeating units of some pathogenic bacterial surface polysaccharides.^1−3^ One of the most common Sia modifications is *O*-acetylation, which has been found at C4, 7, 8, and/or 9 of Sia.^4−6^ The frequencies and patterns of the *O*-acetylation have been reported to vary across species, and specific *O*-acetylation has been shown to change the recognition by some Sia-binding proteins.^2,3,7−11^ However, it remains unclear how *O*-acetylation at different positions in Sias affects the recognition by sialidases that cleave Sias.

Sialidases are a large family of enzymes that are present in organisms ranging from humans to bacteria and viruses. Sialidases encoded by the respiratory pathogen influenza A virus (IAV) are commonly referred to as neuraminidases (NA or N). IAV NAs are one of the viral surface glycoproteins. NAs facilitate IAV movement in human or animal hosts by cleaving the sialic acid from sialoside receptors in mucus or on the infected cell surface that can be bound by the other IAV surface antigen hemagglutinin (HA).^12^ Because of the central role of Sias in IAV infection, host-dependent variations in both the sialyl linkage^13^ and Sia modifications^4^ are believed to be part of the biological barriers limiting the capability of IAVs to replicate, adapt, and spread in new hosts.^14^

Both NA and HA recognize *N*-acetylneuraminic acid (Neu5Ac, Figure 1a), which is the most common Sia form.^6^ However, the specificity of NA for different sialyl linkages and other Sia forms^15,16^ has received little attention as most studies have focused on Sia binding and cleavage using reporter substrates that carry Neu5Ac devoid of *O*-acetylation modifications or sialyl-glycan linkages. The few studies examining Sia modifications have shown that *O*-acetylation can block or slow down Sia cleavage in a sialidase-dependent manner.^2,3,8−11^ For instance, bacterial and human sialidases have been shown to be unable to cleave Neu5Ac modified with a 4-*O*-acetyl group (4-*O*-acetylneuraminic acid or Neu4,5Ac_2_), whereas IAV NAs can readily cleave Neu4,5Ac_2_ from α2–3-sialosides.^16^ Conversely, sialosides containing a terminal 9-*O*-acetylated or 9-*N*-acetylated Sia were shown to be suitable substrates for numerous bacterial sialidases, such as those from *Arthrobacter ureafaciens*, *Clostridium perfringens*, *Streptococcus pneumoniae* (SpNanA/B/C), or *Salmonella typhimurium*, but not human NEU2 or some other bacterial sialidases.^17,18^

**Figure 1:**
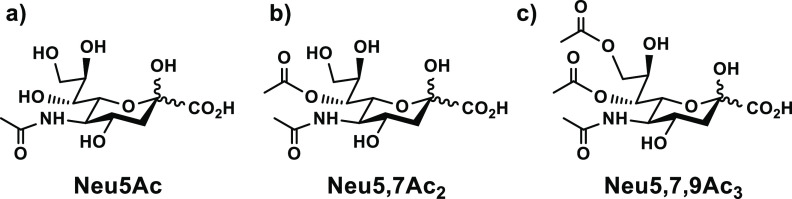
Structures of (a) *N*-acetylneuraminic acid (Neu5Ac), (b) 7-*O*-acetyl Neu5Ac (Neu5,7Ac_2_), and (c) 7,9-di-*O*-acetyl Neu5Ac (Neu5,7,9Ac_3_).

Neu5,9Ac_2_ has not been extensively studied despite being broadly detected in different cells and tissues, and studies for the less common 7-*O*-acetyl Neu5Ac (Neu5,7Ac_2_, Figure 1b) have been even rarer.^7,11^ Neu5,7Ac_2_ and related sialosides are difficult to obtain in high purity and are not stable under physiological conditions because of *O*-acetyl migration and the susceptibility of the *O*-acetyl to cleavage by esterases.^7,19,20^ In fact, mono-*O*-acetylated Sias usually exist as a mixture of Neu5,7Ac_2_, Neu5,8Ac_2_, and Neu5,9Ac_2_.^21,22^ These issues have been overcome in studies on Neu5,7Ac_2_ by using commercially available bovine submaxillary mucin, which presents 7,9-di-*O*-acetyl Neu5Ac (Neu5,7,9Ac_3_, Figure 1c),^4,6,7,23^ but this substrate does not provide information on interactions that are specific to Neu5,7Ac_2_.

Structurally defined synthetic sialosides^24−26^ are indispensable reagents to elucidate the functional roles of naturally occurring *O*-acetyl Sias (OAc-Sias). We have minimized the complications of investigating labile OAc-Sias by previously showing that sialosides containing *N*-acetylated Sias are stable mimics of those with *O*-acetyl Sias and are important tools to explore the biological functions of Sia *O*-acetylation.^19,22,27−29^ Recently, we reported a chemoenzymatic synthon strategy to synthesize 7-*N*-acetyl Neu5Ac (Neu5Ac7NAc) and 7,9-di-*N*-acetyl Neu5Ac (Neu5Ac7,9diNAc)-containing sialosides as stable mimics of their *O*-acetyl-Neu5Ac counterparts.^28^ Here, we show that the chemoenzymatic synthon strategy^28,30^ works well for generating α2–3- and α2–6-sialylated *para*-nitrophenyl β-galactoside (Galβ*p*NP) probes containing Neu5Ac7NAc or Neu5Ac7,9diNAc. These stable mimics of sialosides containing Neu5,7Ac_2_ or Neu5,7,9Ac_3_ are effective reagents to determine the substrate specificity of sialidases from different origins in a microtiter-plate-based high-throughput screening platform.^15,17,18^

The azido-containing six-carbon mannose-based precursors 2,4-diazido-2,4-dideoxy-d-mannose (Man2,4diN_3_, **1**) and 2,4,6-triazido-2,4,6-trideoxy-d-mannose (Man2,4,6triN_3_, **2**) were synthesized from d-galactose (Gal) via an eight-step reaction process.^28^ These precursors were then used as chemoenzymatic synthons for generating α2–3- and α2–6-linked sialosides from *para*-nitrophenyl β-galactoside (Galβ*p*NP, **5**) (Scheme 1) by a one-pot three-enzyme (OP3E) sialylation reaction system. In this system, *Pasteurella multocida* sialic acid aldolase (PmAldolase)^31^ catalyzed the aldol addition reaction of Man2,4diN_3_ (**1**) or Man2,4,6triN_3_ (**2**) with sodium pyruvate to form Neu5,7diN_3_ (**3**) or Neu5,7,9triN_3_ (**4**). The resulting azido-Sia derivatives **3** and **4** were activated by *Neisseria meningitidis* CMP-sialic acid synthetase (NmCSS),^32^ and transferred to Galβ*p*NP (**5**) by a sialyltransferase to form azido-sialoside derivatives **6**–**9**. *Pasteurella multocida* α2–3-sialyltransferase 1 (PmST1)^33^ was used to form α2–3-linked sialosides containing either diazido groups Neu5,7diN_3_α3Galβ*p*NP (**6**) or triazido groups Neu5,7,9triN_3_α3Galβ*p*NP (**7**), whereas *Photobacterium damselae* α2–6-sialyltransferase (Pd2,6ST)^34^ was used to form analogous α2–6-linked sialosides Neu5,7diN_3_α6Galβ*p*NP (**8**) or Neu5,7,9triN_3_α6Galβ*p*NP (**9**). As shown in Table 1, Galβ*p*NP (**5**) was a suitable acceptor for the sialyltransferase in the OP3E sialylation system, and the target sialosides (**6**–**9**) were obtained in yields ranging from 61% to 76%.

**Table 1:**
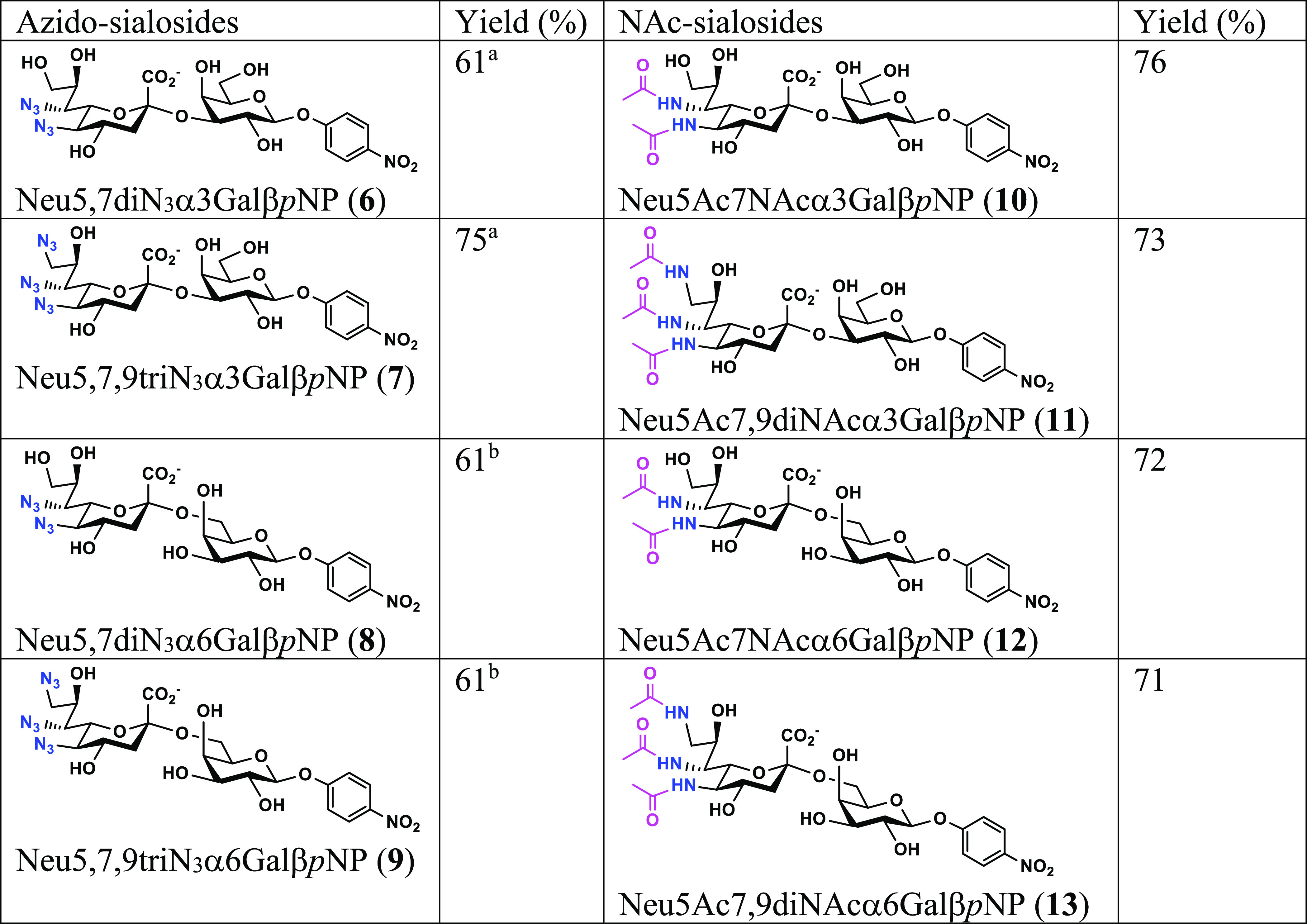
Structures and Yields of the Synthesized N_3_- and NAc-Containing Sialosides

a PmST1 was used as the α2–3sialyltransferase.

b Pd2,6ST was used as the α2–6sialyltransferase.

**Scheme 1:**
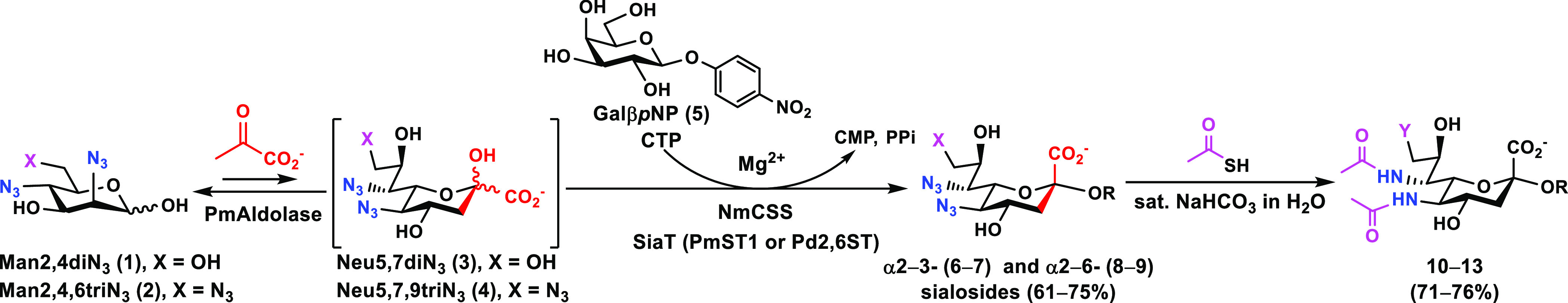
One-Pot Three-Enzyme (OP3E) Synthesis of α2–3 (**6**–**7**)- and α2–6 (**8**–**9**)-Linked Sialosides from Man2,4diN_3_ (**1**) or Man2,4,6triN_3_ (**2**) Followed by Chemical Conversion of Azido Groups to *N*-Acetyl Groups Azido groups in sialosides containing Neu5,7diN_3_ (**6** and **8**) or Neu5,7,9triN_3_ (**7** and **9**) were chemically converted to *N*-acetyl moieties to generate sialosides containing Neu5Ac7NAc (**10** and **12**) or Neu5Ac7,9diNAc (**11** and **13**).

The azido groups in sialosides **6**–**9** were converted to *N*-acetyl groups (Scheme 1) by treating the compound with thioacetic acid in saturated sodium bicarbonate aqueous solution, as previously reported.^28^ The resulting NAc-sialosides (**10**–**13**) were obtained in 71–76% yields (Table 1).

The sialosides containing azido-Sia (**6**–**9**) or NAc-Sia (**10**–**13**) were used together with sialosides containing Neu5Ac (Neu5Acα3Galβ*p*NP and Neu5Acα6Galβ*p*NP)^17^ or Neu5Ac7N_3_ (Neu5Ac7N_3_α3Galβ*p*NP and Neu5Ac7N_3_α6Galβ*p*NP)^35^ that we synthesized previously to examine the substrate specificity of one human and eight bacterial sialidases (Figure 2). The high-throughput colorimetric assays^17^ were carried out in a 384-well plate using the human cytosolic sialidase (hNEU2)^36^ and bacterial sialidases from *Arthrobacter ureafaciens* (Au Sialidase), *Clostridium perfringens* (CpNanI), *Vibrio cholerae* (Vc Sialidase), *Streptococcus pneumoniae* (SpNanA,^37^ SpNanB,^38^ and SpNanC^39^),^40^ and *Bifidobacterium infantis* (BiNanH2).^41^*Pasteurella multocida* α2–3-sialyltransferase 1 (PmST1) was included in the assay because it also has α2–3-sialidase activity in the presence of CMP.^33^

**Figure 2:**
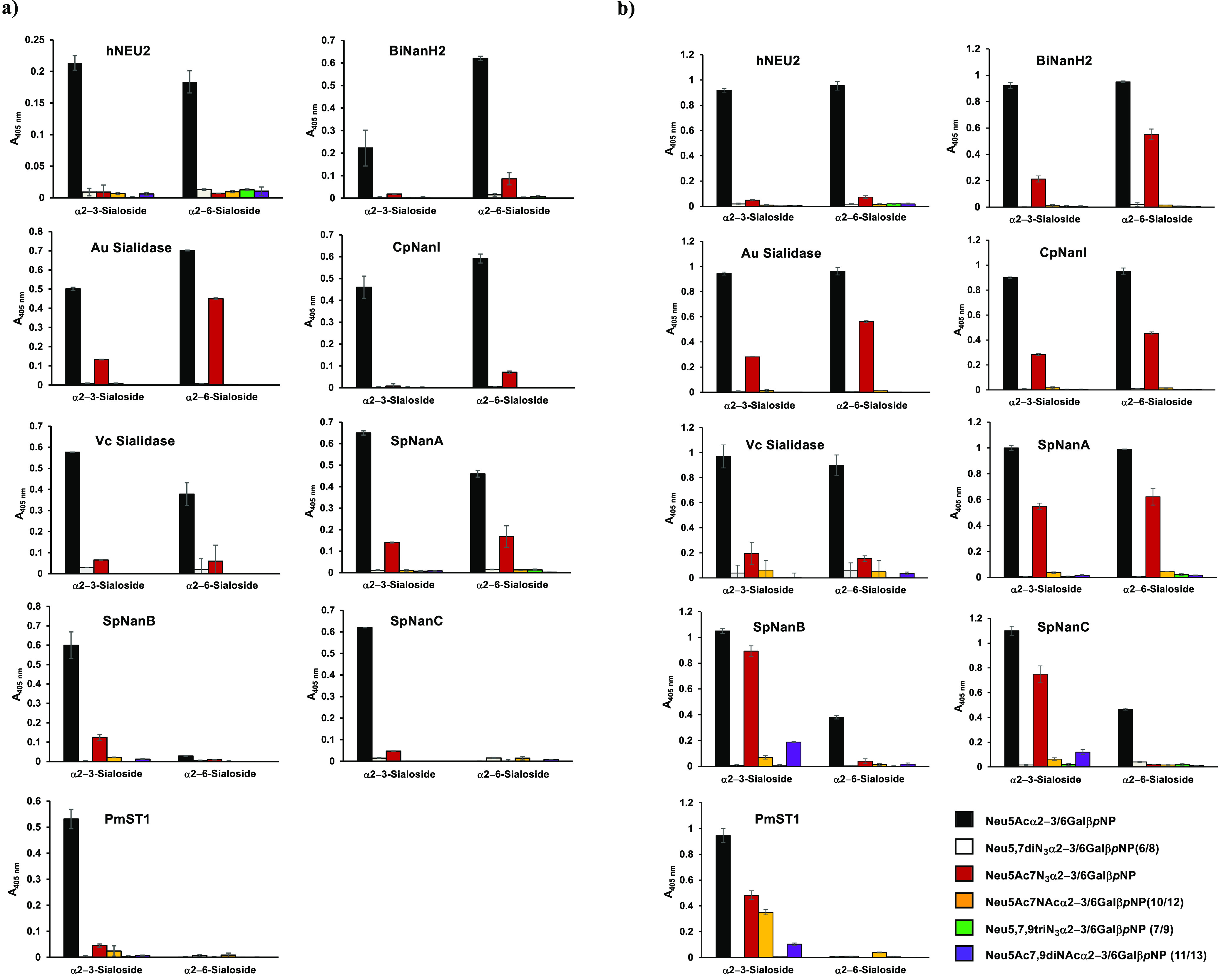
Human and bacterial sialidase substrate specificity studies using sialyl Galβ*p*NPs with low (a) and high (b) enzyme concentrations. Sialidase amounts for the low-concentration assays were standardized using the substrate Neu5Acα3Galβ*p*NP. Sialidase amounts for the high-concentration enzyme assays were 4–500 fold (hNEU2, 8-fold; BiNanH2, 10-fold; Au Sialidase, 5-fold; CpNanI, 17-fold; Vc Sialidase, 4-fold; SpNanA, 100-fold; SpNanB, 100-fold; SpNanC, 500-fold; PmST1, 20-fold) higher than those used in the low-concentration enzyme assays depending on the availability of the enzymes. Abbreviations: h, human; Bi, *Bifidobacterium infantis*; Au, *Arthrobacter ureafaciens*; Cp, *Clostridium perfringens*; Vc, *Vibrio cholerae*; Sp, *Streptococcus pneumoniae*; PmST1, the sialidase activity of *Pasteurella multocida* α2–3-sialyltransfearse 1 in the presence of CMP (0.4 mM).

The relative preference of the sialidases toward different substrates were compared by standardizing the low enzyme concentration amounts on the basis of their ability to process the substrate Neu5Acα3Galβ*p*NP and produce an *A*_405 nm_ of less than 0.7 in the assay (Figure 2a). Depending on enzyme availability, 4–500-fold higher amounts were used for the high sialidase concentration assays (Figure 2b). Each sialidase was incubated with the different sialoside probes in the presence of an excess amount of β-galactosidase at 37 °C for 30 min. Directly after the incubation, the pH of the reaction was adjusted to >9.5 using *N*-cyclohexyl-3-aminopropanesulfonic acid (CAPS) buffer, and the *A*_405 nm_ was recorded. In this assay, the β-galactosidase produced *para*-nitrophenol only if the sialoside probe was cleaved into a galactoside by the sialidase.^15,17,18,36^

Results with both low and high sialidase amounts showed that sialosides containing a C7-azido derivative of Neu5Ac (Neu5Ac7N_3_α3Galβ*p*NP and/or Neu5Ac7N_3_α6Galβ*p*NP) were recognized and cleaved by all bacterial sialidases tested, but not hNEU2 (Figure 2a,b). In contrast, the α2–3-linked sialoside containing a C7-NAc-substituted Neu5Ac (Neu5Ac7NAcα3Galβ*p*NP, **10**) was only weakly tolerated by the α2–3-sialidase activity of the multifunctional PmST1 when a 20-fold higher enzyme concentration was used. Neu5,7diN_3_α3/6Galβ*p*NP (**6** and **8**) and Neu5,7,9triN_3_α3/6Galβ*p*NP (**7** and **9**) were resistant to cleavage by all bacterial and human sialidases tested, which indicates the importance of the acetamido group at the C5 of Neu5Ac for recognition by these sialidases. Neu5Ac7,9diNAcα3Galβ*p*NPs (**11**) and the α2–6-linked sialosides Neu5Ac7NAcα6Galβ*p*NP (**12**) and Neu5Ac7,9diNAcα6Galβ*p*NPs (**13**) were also largely resistant to cleavage by the human and bacterial sialidases tested (Figure 2b). The lone exception was Neu5Ac7,9diNAcα6Galβ*p*NPs (**13**), where very weak sialidase activity was observed only for the high enzyme concentrations of SpNanB, SpNanC, and PmST1.

These data indicate that the recombinant hNEU2 and commercially obtained Vc sialidase had either no or very low tolerance toward modifications at C7 of Neu5Ac with or without additional C5-azido substitution and/or C9-modification in the sialoside substrates. It is worth noting that SpNanB and SpNanC, which were reported as having specificity for α2–3-sialyl linkages,^40^ showed some ability to cleave the α2–6-sialyl linkage in Neu5Acα6Galβ*p*NP when very high enzyme concentrations (100–500-fold) were used (Figure 2b), whereas PmST1 sialidase activity retained its α2–3-sialyl linkage selectivity even at rather high (20-fold) enzyme concentrations. The protective effects of Sia *O*-acetylation against sialidase cleavage have previously been reported;^42^ however, only a limited number of sialidases have been shown to be incapable of cleaving sialosides containing Neu5,9Ac_2_ or its 9-*N*-acetyl analogue Neu5Ac9NAc.^18^ The results obtained here demonstrate that most bacterial and human sialidases cannot recognize or cleave sialoglycans containing Neu5Ac7NAc or Neu5Ac7,9diNAc and, likely, their naturally occurring *O*-acetyl counterparts. The substitution of Neu5Ac C7-OH in sialosides with a group larger than N_3_ most likely blocks the sialoside recognition by the human and bacterial sialidases tested.

The α2–3-sialidase activity of PmST1 is mainly attributed to its reversible α2–3-sialyltransferase activity.^43,44^ Interestingly, the α2–3-sialyltransferase activity of PmST1 can effectively use CMP-activated Neu5,7diN_3_ and Neu5,7,9triN_3_ generated *in situ* during the OP3E reaction system as donor substrates for synthesizing the previously reported α2–3/6-linked sialosides^28^ and compounds **6**–**9** shown in Table 1. However, the α2–3-sialidase activity of PmST1 was not detectable for the α2–3-linked Neu5,7diN_3_- and Neu5,7,9triN_3_-sialosides, which indicates that the equilibrium of the reverse sialylation reaction of PmST1 was impacted by substrate modification.

The same set of sialyl Galβ*p*NP probes were used together with five additional sialosides that we synthesized previously to examine the substrate specificity of NAs from several IAVs. In addition to the sialosides containing Neu5Ac (Neu5Acα3Galβ*p*NP and Neu5Acα6Galβ*p*NP)^17^ or Neu5Ac7N_3_ (Neu5Ac7N_3_α3Galβ*p*NP and Neu5Ac7N_3_α6Galβ*p*NP)^35^ described above, Neu5Ac9NAc-containing sialosides Neu5Ac9NAcα3Galβ*p*NP and Neu5Ac9NAcα6Galβ*p*NP,^18^ as well as sialosides that contain a 9-deoxy-derivative of Neu5,7diN_3_ (Leg5,7diN_3_α3Galβ*p*NP and Leg5,7diN_3_α6Galβ*p*NP) and 9-deoxy-derivative of Neu5Ac7NAc (Leg5,7diNAcα3Galβ*p*NP and Leg5,7diNAcα6Galβ*p*NP)^30^ were also used as probes. The analysis was performed with NAs of subtype 1 (N1) and 2 (N2) from the following strains: A/Victoria/2570/2019 (N1-Vic19), A/Brisbane/02/2018 (N1-BR18), A/Darwin/9/2021 (N2-Dar21), and A/Kansas/14/2017 (N2-Kan17). The low-IAV NA concentration analysis (Figure 3a) was performed using 0.08–0.2 μg/well, and the high-concentration analysis (Figure 3b) was performed using 10-fold higher amounts. Similar to most bacterial sialidases tested (Figure 2b and previous results^18,30^), the NAs catalyzed cleavage of both Neu5Ac7N_3_ and Neu5Ac9NAc (Figure 3). In contrast to the bacterial sialidases, the NAs also readily cleaved sialosides containing Neu5Ac7NAc, especially at high enzyme concentrations (Figure 3). This observation suggests that the naturally occurring Neu5,9Ac_2_- and Neu5,7Ac_2_-sialosides are potential substrates for NAs from IAVs, especially during replication when the concentration of NA in the local environment is high. Supporting the observed tolerance to C7 and C9-modifications of Neu5Ac in the sialoside substrate, the IAV NAs that were tested also displayed weak activity toward Neu5Ac7,9diNAc and Leg5,7diNAc (Figure 3b).

**Figure 3:**
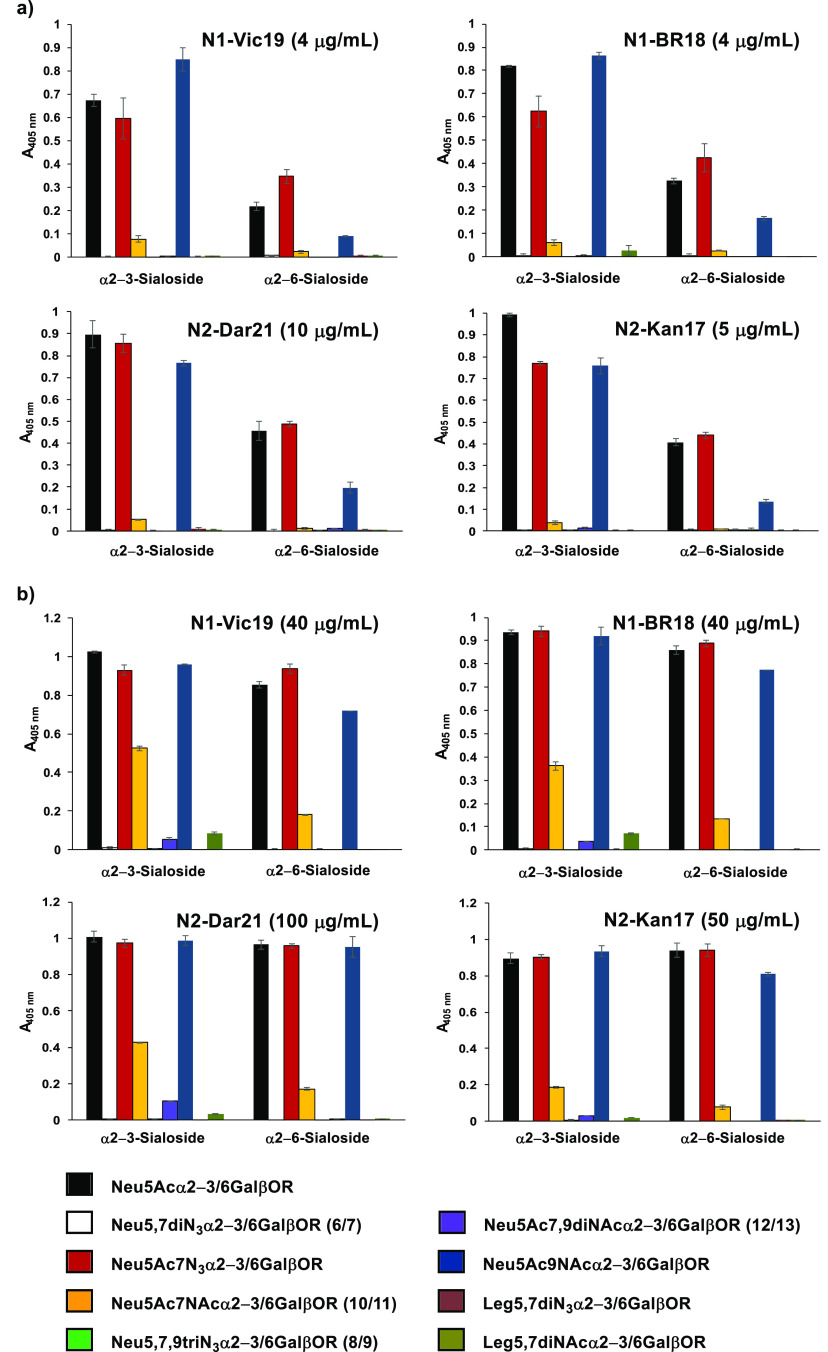
Substrate specificity analysis of IAV NAs using Siaα3/6Galβ*p*NP and derivatives with low (a) and 10-fold higher (b) enzyme concentrations, respectively.

The combined results indicate that Neu5Ac7NAc-containing sialosides can be used as selective substrates for NAs from IAVs. This can be explained by the crystal structures of NA complexed with the sialic acid analog zanamivir where its C7-hydroxyl is freely exposed to water in the NA substrate binding pocket^45^ and is supported by the observation that NA transition state inhibitors containing C7-OMe,^46^ C7-*O*-carbamate,^47^ and other C7-derivatives^48,49^ do not exhibit significantly reduced inhibition activity.

In conclusion, we demonstrate that Man2,4diN_3_ and Man2,4,6triN_3_ are well suited chemoenzymatic synthons for generating stable *p*NP-tagged α2–3- and α2–6-linked sialyl glycosides containing Neu5Ac7NAc and Neu5Ac7,9diNAc, which structurally mimic those with naturally occurring *O*-acetyl Sias Neu5,7Ac_2_ and Neu5,7,9Ac_3_, respectively. The approach of combining the OP3E sialylation system with the chemical conversion was critical for synthesizing the Neu5Ac7NAc-containing sialosides that were found to be selective substrates for IAV NAs. It also effectively produced a set of new probes for analyzing the substrate specificity of a large family of sialidases in a high-throughput screening format.

## Methods

### General Methods

Chemicals were purchased and used without further purification. Nuclear magnetic resonance (NMR) spectra were recorded in the NMR facility of the University of California, Davis on a 800 MHz Bruker Avance III-NMR spectrometer or a 400 MHz Bruker Avance III HD Nanobay spectrometer. Chemical shifts are reported in parts per million (ppm) on the δ scale. High-resolution electrospray ionization (ESI) mass spectra were obtained using a Thermo Scientific Q Exactive HF Orbitrap Mass Spectrometer at the mass spectrometry facility in the University of California, Davis. Column chromatography was performed using a CombiFlash Rf 200i system with either Redi*Sep* Rf silica columns or an ODS-SM (C18) column (51 g, 50 μm, 120 Å, Yamazen) or manually using columns packed with silica gel 60 Å (230–400 mesh, Sorbent Technologies). Thin-layer chromatography (TLC) was performed on silica gel plates (Sorbent Technologies) using anisaldehyde sugar stain or 5% sulfuric acid in ethanol stain for detection. Gel filtration chromatography was performed with a column (100 cm × 2.5 cm) packed with Bio-Gel P-2 Fine resins (Bio-Rad). PmAldolase,^31^ NmCSS,^32^ PmST1,^33^ and Pd2,6ST^34^ were expressed and purified as described previously. Man2,4diN_3_ (**1**) and Man2,4,6triN_3_ (**2**) were synthesized as we previously reported.^28^

#### General Procedures for One-Pot Three-Enzyme (OP3E) Preparative-Scale Synthesis of Neu5,7diN_3_**α**2–3/6-Linked Sialosides (**6** and **8**)

Acceptor Galβ*p*NP (**5**) (50–55 mg, 0.17–0.18 mmol), Man2,4diN_3_ (**1**, 78–83 mg, 0.34–0.36 mmol), sodium pyruvate (180–198 mg, 1.7–1.8 mmol), and CTP (260–290 mg, 0.5–0.550 mmol) were dissolved in water in a 50 mL centrifuge tube containing Tris-HCl buffer (100 mM, pH 8.5) and MgCl_2_ (20 mM). After the addition of PmAldolase (2–3 mg), NmCSS (0.5 mg), and a sialyltransferase PmST1 (2–3 mg) or Pd2,6ST (3–4 mg), water was added to bring the final concentration of Man2,4diN_3_ (**1**) to 10 mM. The reaction mixture was incubated at 30 °C for 24–36 h. The reaction progress was monitored using TLC (EtOAc/MeOH/H_2_O = 6:1:1, by volume) and mass spectrometry. The reaction mixture was diluted with the same volume of ethanol and incubated at 4 °C for 30 min. The resulting mixture was centrifuged. The supernatant was concentrated and purified by a CombiFlash Rf 200i system using a C18 column (CH_3_CN in H_2_O gradient as eluant) to produce Neu5,7diN_3_α2–3Galβ*p*NP (**6**) (60 mg, 61% yield) or Neu5,7diN_3_α2–6Galβ*p*NP (**8**) (68 mg, 61% yield) as a white solid.

#### General Procedures for One-Pot Three-Enzyme (OP3E) Preparative Scale Synthesis of Neu5,7,9triN_3_α2–3/6-Linked Sialosides (**7** and **9**)

Acceptor Galβ*p*NP (**5**) (45–50 mg, 0.15–0.17 mmol), Man2,4,6triN_3_ (**2**, 76–84 mg, 0.3–0.35 mmol), sodium pyruvate (165–190 mg, 1.5–1.75 mmol), and CTP (237–277 mg, 0.45–0.53 mmol) were dissolved in water in a 50 mL centrifuge tube containing Tris-HCl buffer (100 mM, pH 8.5) and MgCl_2_ (20 mM). After the addition of PmAldolase (2–3 mg), NmCSS (0.5 mg), and a sialyltransferase PmST1 (2–3 mg) or Pd2,6ST (3–4 mg), water was added to bring the final concentration of Man2,4,6triN_3_ (**2**) to 10 mM. The reaction was then carried out, and the products were purified similarly to that described above for compounds **6** and **8** to obtain Neu5,7,9triN_3_α2–3Galβ*p*NP (**7**) (78 mg, 75% yield) or Neu5,7,9triN_3_α2–6Galβ*p*NP (**9**) (59 mg, 61% yield) as a white solid.

#### General Procedures for Converting Azido-Containing Glycosides (**6**–**9**) to *N*-Acetyl-Containing Glycosides (**10**–**13**)

An azido-containing glycoside (35–40 mg) was added to a saturated sodium bicarbonate solution in water (1–2 mL) in a round-bottom flask (25 mL). Thioacetic acid (50–100 μL, 12–24 equiv) was then added drop-by-drop under argon at room temperature. The reaction mixture was stirred at 70 °C for 20 h when TLC analysis (EtOAc/MeOH/H_2_O = 10:2:1, by volume) indicated the completion of the reaction. The solvent was then removed under vacuum, and the residue was passed through a Bio-Gel P-2 gel filtration (water was used as an eluent). The fractions containing the product were combined and concentrated. The resulting mixture was further purified by a silica gel chromatography using a mixed solvent (ethyl acetate/methanol/water = 10:1:0.1, by volume) as an eluent, followed by a C18-column purification (CH_3_CN in H_2_O gradient was used as running solvent) to obtain pure product as a white sold: Neu5Ac7NAcα2–3Galβ*p*NP (**10**) (30 mg, 76% yield), Neu5Ac7,9diNAcα2–3Galβ*p*NP (**11**) (31 mg, 73% yield), Neu5Ac7NAcα2–6Galβ*p*NP (**12**) (28 mg, 72% yield), or Neu5Ac7,9diNAcα2–6Galβ*p*NP (**13**) (27 mg, 71% yield).

### Bacterial Sialidase and Human NEU2 Substrate Specificity Studies

Substrate specificity assays were carried out in duplicate using 384-well plates. The final reaction volume was 20 μL and contained a sialoside (0.3 mM) and β-galactosidase (12 μg) with or without a sialidase in a buffer solution. Reactions without a sialidase were used as negative controls and for background readings. The reactions were incubated for 30 min at 37 °C and were stopped by adding 40 μL of 0.5 M CAPS buffer (pH 10.5) to each well. The amount of the *para*-nitrophenolate formed was determined by measuring the *A*_405 nm_ of the reaction mixtures using a microplate reader. Sialidasae substrate specificities were carried out at a high concentration of sialidases (Figure 2a) and a low concentration of enzymes (Figure 2b) to adjust the absorbance at 405 nm below 0.7. The assay conditions for low and high concentrations of different sialidases are described below with enzyme amounts presented as (low/high, #-fold difference): hNEU2 (0.1 μg/0.8 μg, 8-fold difference), MES buffer (100 mM, pH 5.0); BiNanH2 (0.6 μg/6 μg, 10-fold difference), NaOAc buffer (100 mM, pH 5.0); AuSialidase (0.4 mU/2 mU, 5-fold difference), NaOAc buffer (100 mM, pH 5.5); CpNanI (0.6 mU/10 mU, 17-fold difference), MES buffer (100 mM, pH 5.0); VcSialidase (0.5 mU/2 mU, 4-fold difference), NaCl (150 mM), CaCl_2_ (10 mM), NaOAc buffer (100 mM, pH 5.5); SpNanA (14 ng/1.4 μg, 100-fold difference), NaOAc buffer (100 mM, pH 6.0); SpNanB (0.08 μg/8 μg, 100-fold difference), NaOAc buffer (100 mM, pH 6.0); SpNanC (0.04 μg/20 μg, 500-fold difference), MES buffer (100 mM, pH 6.5); and PmST1 (0.75 μg/15 μg, 20-fold difference), CMP (0.4 mM), NaOAc buffer (100 mM, pH 5.5).

### Recombinant Influenza NA Protein Production and Purification

Baculoviruses (BVs) encoding secreted N1-BR18 and N2-Kan17 were produced by Genscript. Both constructs included a signal peptide (GP67a), 6 × His-tag, tetrabrachion tetramerization domain, and a seven-residue linker followed by either N1 residues 82–469 or N2 residues 74–469 from the respective IAV strains A/Brisbane/02/2018 (H1N1) or A/Kansas/14/2017 (H3N2). Sf9 cells were grown and infected, as previously described.^50^ Culture medium was clarified by two sedimentations (10 min; 4000*g* and 30 min; 10 000*g*), which was concentrated 6-fold by tangential flow filtration (TFF) using a 30 kDa molecular weight cutoff (MWCO) capsule (Pall), and diafiltrated (5 volumes) into Binding buffer (50 mM Tris-HCl, pH 7.0, 300 mM NaCl, 1 mM CaCl_2_, 30 mM imidazole pH 7.0). Samples were loaded onto a 1 mL HisTrap crude FF column (Cytiva) using an AKTA Start, washed using 10 column volumes (CVs) of binding buffer, and eluted with a 20 CV linear imidazole gradient from 30–500 mM. NA fractions were pooled and concentrated using a 30 kDa MWCO centrifugal filter (Amicon). Secreted N1-Vic19 and N2-Dar21 BVs were produced by the Bac-to-Bac Baculovirus Expression system (Thermo Fisher) using constructs containing a signal peptide (azurocidin), Strep-Tag, tetrabrachion tetramerization domain, and a seven-residue linker followed by N1 residues 35–469 or N2 residues 74–469 from the respective strains A/Victoria/2570/2019 (H1N1) or A/Darwin/9/2021 (H3N2). Expression and TFF were performed identically without imidazole. Diafiltrated samples were purified by Strep-Tactin XT affinity chromatography (Cytiva) according to the manual (iba). NA fractions were pooled, mixed with 9 volumes of Buffer A (30 mM MES pH 6.5, 1 mM CaCl_2_), loaded onto a 1 mL SP-sepharose column, and washed with 10 CVs of Buffer A. For N1-Vic19, an additional wash with 10 CVs of Buffer A containing 200 mM NaCl was performed prior to elution with Buffer A containing 300 mM NaCl. For N2-Dar21, an additional wash with 10 CVs of Buffer A containing 50 mM NaCl was performed prior to elution with Buffer A containing 150 mM NaCl. All purified NAs were dialyzed 3 times at 4 °C against 1 L of buffer (50 mM Tris pH 6.5, 150 mM NaCl, 1 mM CaCl_2_) using a 10 kDa MWCO cassette (Thermo Scientific). Protein concentrations were determined by *A*_280 nm_ and adjusted to ∼0.5–1.0 mg/mL prior to aliquoting and storage at −80 °C.

### IAV NA Substrate Specificity Studies

The substrate specificity assays were carried out in duplicate in a 384-well plate using a final volume of 20 μL. Each sialoside (0.3 mM) was incubated with an NA and an excess amount of β-galactosidase (12 μg) in MES buffer (25 mM, pH 6.0) containing NaCl (150 mM) and CaCl_2_ (1 mM) at 37 °C for 30 min. Assays were stopped with 40 μL of 0.5 M CAPS buffer (pH 11.5), and *A*_405 nm_ readings were obtained by a microplate reader. For every sialoside tested, duplicate reactions without a sialidase were used as negative controls and for background readings. The NA amounts for low-concentration assays were: N1-Vic19 (0.08 μg), N1-BR18 (0.08 μg), N2-Dar21 (0.20 μg), and N2-Kan17 (0.10 μg). Amounts 10-fold higher were used for high-NA concentration assays.

## Supporting Information Available

NMR chemical shifts, high-resolution mass spectrometry (HRMS) data, and NMR spectra of sialoside products **6**–**13** (PDF)
