# Supplementary material for: Sialosides Containing 7-N-Acetyl Sialic Acid Are Selective Substrates for Neuraminidases from Influenza A Viruses
Source: ACS Infect Dis. 2022 Dec 1;9(1):33–41. doi: 10.1021/acsinfecdis.2c00502 (PMC9840695; doi:10.1021/acsinfecdis.2c00502)

Supporting Information

Sialosides Containing 7-*N*-Acetyl Sialic Acid Are Selective Substrates for Neuraminidases from Influenza A Virus

Anoopjit Singh Kooner,[a] Yue Yuan,[a] Hai Yu,[a] Hyeog Kang,[b] Laura Klenow,[b] Robert Daniels,[b] and Xi Chen[a,]*

[a]Department of Chemistry, University of California, Davis, California, 95616, United States

[b]Division of Viral Products, Center for Biologics Evaluation and Research, Food and Drug Administration, Silver Spring, MD 20993, United States

*Corresponding author: xiichen@ucdavis.edu

Table of Contents

^{1}H and ^{13}C NMR chemical shifts and HRMS data for sialosides **6–13**…………………………....S2–S3

^{1}H and ^{13}C NMR spectra of sialosides **6–13**…………………………………….....................S4–S11

^{1}H and ^{13}C NMR chemical shifts, and HRMS data for sialosides **6–13**

Neu5,7diN$_3$α2–3Galβ*p*NP (6): ^{1}H NMR (800 MHz, D_2O) δ 8.29–8.27 (m, 2H), 7.27–7.25 (m, 2H), 5.30 (d, *J* = 7.8 Hz, 1H), 4.18 (ddd, *J* = 9.8, 3.2, 1.1 Hz, 1H), 4.09–4.06 (m, 1H), 4.03 (d, *J* = 2.9 Hz, 1H), 3.92–3.89 (m, 2H), 3.76–3.69 (m, 4H), 3.65–3.63 (m, 1H), 3.62–3.59 (m, 1H), 3.22–3.19 (m, 2H), 2.77 (dd, *J* = 12.7, 4.8 Hz, 1H), 1.93 (t, *J* = 12.3 Hz, 1H). ^{13}C NMR (200 MHz, D_2O) δ 173.4, 161.7, 142.5, 126.1, 116.4, 100.5, 99.6, 75.6, 75.4, 72.3, 70.4, 69.5, 68.7, 67.6, 63.2, 62.5, 60.6, 60.3, 42.5. HRMS (ESI-Orbitrap) *m/z* calculated for $C_{21}H_{26}N_7O_{14}^-$ [M-H]$^-$ 600.1543, found 600.1587.

Neu5,7diN$_3$α2–6Galβ*p*NP (8): ^{1}H NMR (800 MHz, D_2O) δ 8.32–8.28 (m, 2H), 7.29–7.24 (m, 2H), 5.19 (d, *J* = 7.7 Hz, 1H), 4.02 (d, *J* = 3.5 Hz, 1H), 4.00–3.95 (m, 2H), 3.90 (ddd, *J* = 11.8, 8.9, 2.0 Hz, 2H), 3.87–3.83 (m, 1H), 3.78 (ddd, *J* = 10.0, 3.5, 1.1 Hz, 1H), 3.76–3.72 (m, 2H), 3.71–3.66 (m, 3H), 3.46 (t, *J* = 9.8 Hz, 1H), 2.74 (dd, *J* = 9.6, 4.8 Hz, 1H), 1.73 (d, *J* = 12.8 Hz, 1H). ^{13}C NMR (200 MHz, D_2O) δ 173.1, 161.8, 142.5, 126.1, 116.5, 100.6, 99.7, 74.0, 72.3, 72.0, 70.5, 70.3, 69.3, 68.4, 63.5, 63.3, 62.4, 60.8, 39.3. HRMS (ESI-Orbitrap) *m/z* calculated for $C_{21}H_{26}N_7O_{14}^-$ [M-H]$^-$ 600.1543, found 600.1587.

Neu5,7,9triN$_3$α2–3Galβ*p*NP (7): ^{1}H NMR (400 MHz, D_2O) δ 8.29–8.21 (m, 2H), 7.25 (dd, *J* = 9.1, 1.6 Hz, 2H), 5.27 (d, *J* = 7.8 Hz, 1H), 4.25–4.14 (m, 2H), 4.04 (d, *J* = 3.1 Hz, 1H), 3.95–3.86 (m, 2H), 3.77–3.46 (m, 8H), 2.78 (dd, *J* = 12.7, 4.7 Hz, 1H), 1.94 (t, *J* = 12.3 Hz, 1H). ^{13}C NMR (100 MHz, D_2O) δ 173.3, 161.7, 142.5, 126.1, 116.4, 100.5, 99.7, 75.6, 75.4, 72.2, 69.5, 69.2, 68.7, 67.5, 63.2, 61.3, 60.6, 59.3, 53.1, 39.1. HRMS (ESI-Orbitrap) *m/z* calculated for $C_{21}H_{25}N_{10}O_{13}^-$ [M-H]$^-$ 625.1608, found 625.1658.

Neu5,7,9triN$_3$α2–6Galβ*p*NP (9): ^{1}H NMR (400 MHz, D_2O) δ 8.30–8.08 (m, 2H), 7.39–6.88 (m, 2H), 5.10 (d, *J* = 7.6 Hz, 1H), 4.04–3.74 (m, 5H), 3.73–3.55 (m, 6H), 3.48–3.32 (m, 2H), 2.66 (dd, *J* = 12.7, 4.8 Hz, 1H), 1.66 (t, *J* = 12.2 Hz, 1H). ^{13}C NMR (100 MHz, D_2O) δ 173.1, 161.8, 142.5, 126.1, 116.6, 100.6, 99.7, 74.1, 72.3, 71.9, 70.3, 69.3, 68.4, 63.4, 63.3, 61.7, 53.1, 40.0. HRMS (ESI-Orbitrap) *m/z* calculated for $C_{21}H_{25}N_{10}O_{13}^-$ [M-H]$^-$ 625.1608, found 625.1658.

Neu5Ac7NAcα2–3Galβ*p*NP (10): ^{1}H NMR (800 MHz, D_2O) δ 8.35–8.30 (m, 2H), 7.35–7.28 (m, 2H), 5.33 (d, *J* = 7.8 Hz, 1H), 4.31 (dd, *J* = 9.8, 3.2 Hz, 1H), 4.10 (d, *J* = 3.2 Hz, 1H), 4.03 (dd, *J* = 9.6, 3.1 Hz, 1H), 3.98 (dtd, *J* = 16.2, 8.4, 7.9, 4.3 Hz, 3H), 3.93 (dd, *J* = 10.5, 3.0 Hz, 1H), 3.85–3.77 (m, 3H), 3.68 (ddd, *J* = 11.8, 9.7, 4.5 Hz, 1H), 3.50 (dd, *J* = 14.2, 2.7 Hz, 1H), 3.13 (dd, *J* = 14.2, 8.0 Hz, 1H), 2.88 (dd, *J* = 12.5, 4.6 Hz, 1H), 2.05 (s, 3H), 2.01 (s, 3H), 1.99 (s, 3H), 1.87 (t, *J* = 12.1 Hz, 1H). ^{13}C NMR (200 MHz, D_2O) δ 174.0, 173.9, 173.8, 161.7, 142.5, 126.1, 116.4, 99.6, 99.6, 75.5, 75.4, 71.8, 71.7, 68.8, 68.6, 66.8, 62.4, 60.7, 51.8, 49.2, 40.2, 22.1, 21.9. HRMS (ESI-Orbitrap) *m/z* calculated for $C_{25}H_{34}N_3O_{16}^-$ [M-H]$^-$ 632.1945, found 632.1996.

Neu5Ac7,9diNAcα2–3Galβ*p*NP (11): ^{1}H NMR (800 MHz, D_2O) δ 8.35–8.30 (m, 2H), 7.35–7.28 (m, 2H), 5.33 (d, *J* = 7.8 Hz, 1H), 4.31 (dd, *J* = 9.8, 3.2 Hz, 1H), 4.10 (d, *J* = 3.2 Hz, 1H), 4.03 (dd, *J* = 9.6, 3.1 Hz, 1H), 3.98 (dtd, *J* = 16.2, 8.4, 7.9, 4.3 Hz, 3H), 3.93 (dd, *J* = 10.5, 3.0 Hz, 1H), 3.85–3.77 (m, 3H), 3.68 (ddd, *J* = 11.8, 9.7, 4.5 Hz, 1H), 3.50 (dd, *J* = 14.2, 2.7 Hz, 1H), 3.13 (dd, *J* = 14.2, 8.0 Hz, 1H), 2.88 (dd, *J* = 12.5, 4.6 Hz, 1H), 2.05 (s, 3H), 2.01 (s, 3H), 1.99 (s, 3H), 1.87 (t, *J* = 12.1 Hz, 1H). ^{13}C NMR (200 MHz, D_2O) δ 174.2, 174.1, 173.9, 173.5, 161.8, 142.6, 126.2, 116.5, 99.8, 99.7, 75.6, 75.5, 71.9, 69.8, 68.9, 68.6, 66.9, 60.7, 51.9, 50.6, 41.9, 40.1, 22.2, 22.0, 21.9. HRMS (ESI-Orbitrap) *m/z* calculated for $C_{27}H_{37}N_4O_{16}^-$ [M-H]$^-$ 673.2210, found 673.2267.

Neu5Ac7NAcα2–6Galβ*p*NP (12): ^{1}H NMR (400 MHz, D_2O) δ = 1.77 (s, 3H), 1.84 (s, 3H), 1.89 (t, 1H), 2.68–2.74 (m, 1H), 3.34–3.45 (m, 1H), 3.50–3.72 (m, 5H), 3.72–3.84 (m, 3H), 3.85–3.99 (m,

4H), 5.12 (d, J = 7.6 Hz, 1H), 7.22 (d, J = 9.3 Hz, 2H), 8.23 (d, J = 9.3 Hz, 2H). ^{13}C NMR (400 MHz, D_2O) δ = 173.9, 173.8, 173.7, 161.6, 142.4, 126.0, 116.3, 99.5, 99.5, 75.4, 75.3, 71.7, 71.6, 68.7, 68.5, 66.7, 62.3, 60.6, 51.7, 49.1, 40.1, 22.0, 21.8. HRMS (ESI-Orbitrap) *m/z* calculated for $C_{25}H_{34}N_3O_{16}^-$ [M-H]$^-$ 632.1945, found 632.1993.

Neu5Ac7,9diNAcα2–6Galβ*p*NP (13): ^{1}H NMR (800 MHz, D_2O) δ 8.39–8.35 (m, 2H), 7.38–7.34 (m, 2H), 5.25 (dd, J = 7.8, 1.0 Hz, 1H), 4.09 (q, J = 4.1 Hz, 2H), 4.04 (dd, J = 10.5, 8.4 Hz, 1H), 4.01–3.91 (m, 4H), 3.89–3.84 (m, 1H), 3.76–3.72 (m, 1H), 3.70–3.63 (m, 2H), 3.47 (dd, J = 14.3, 2.6 Hz, 1H), 3.09 (dd, J = 14.2, 8.0 Hz, 1H), 2.85 (dd, J = 12.6, 4.0 Hz, 1H), 2.02 (s, 3H), 1.99 (d, J = 0.7 Hz, 3H), 1.95–1.91 (m, 3H), 1.72 (t, J = 11.2, 1H). ^{13}C NMR (200 MHz, D_2O) δ 174.2, 174.0, 173.7, 173.3, 162.0, 142.6, 126.2, 116.5, 100.3, 100.0, 74.2, 72.5, 71.6, 70.4, 69.6, 68.6, 68.6, 63.4, 52.0, 50.6, 41.9, 40.3, 22.2, 21.9, 21.9. HRMS (ESI-Orbitrap) *m/z* calculated for $C_{27}H_{37}N_4O_{16}^-$ [M-H]$^-$ 673.2210, found 673.2267.

^{1}H and ^{13}C NMR spectra of Neu5,7diN$_3$α2–3Galβ*p*NP (**6**)

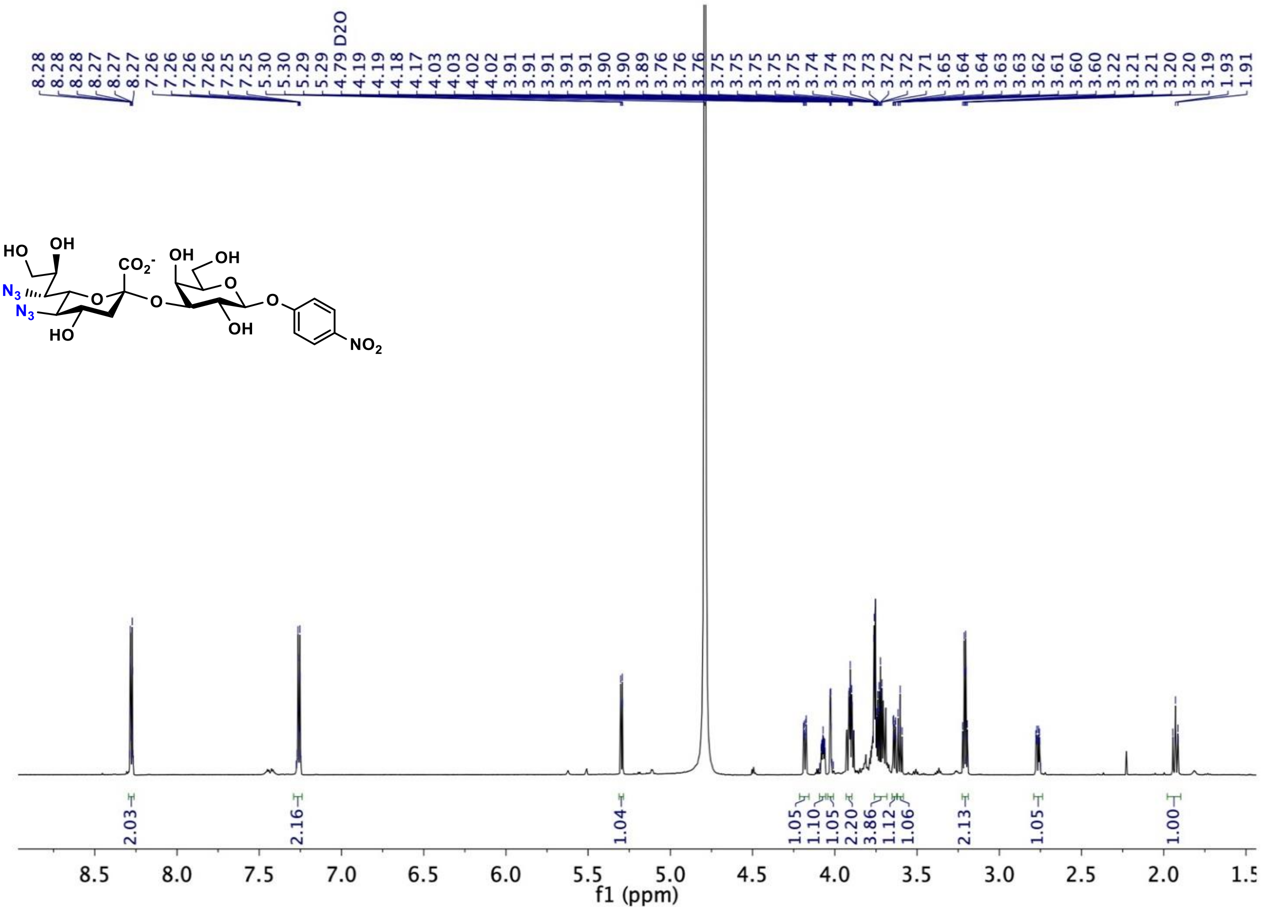

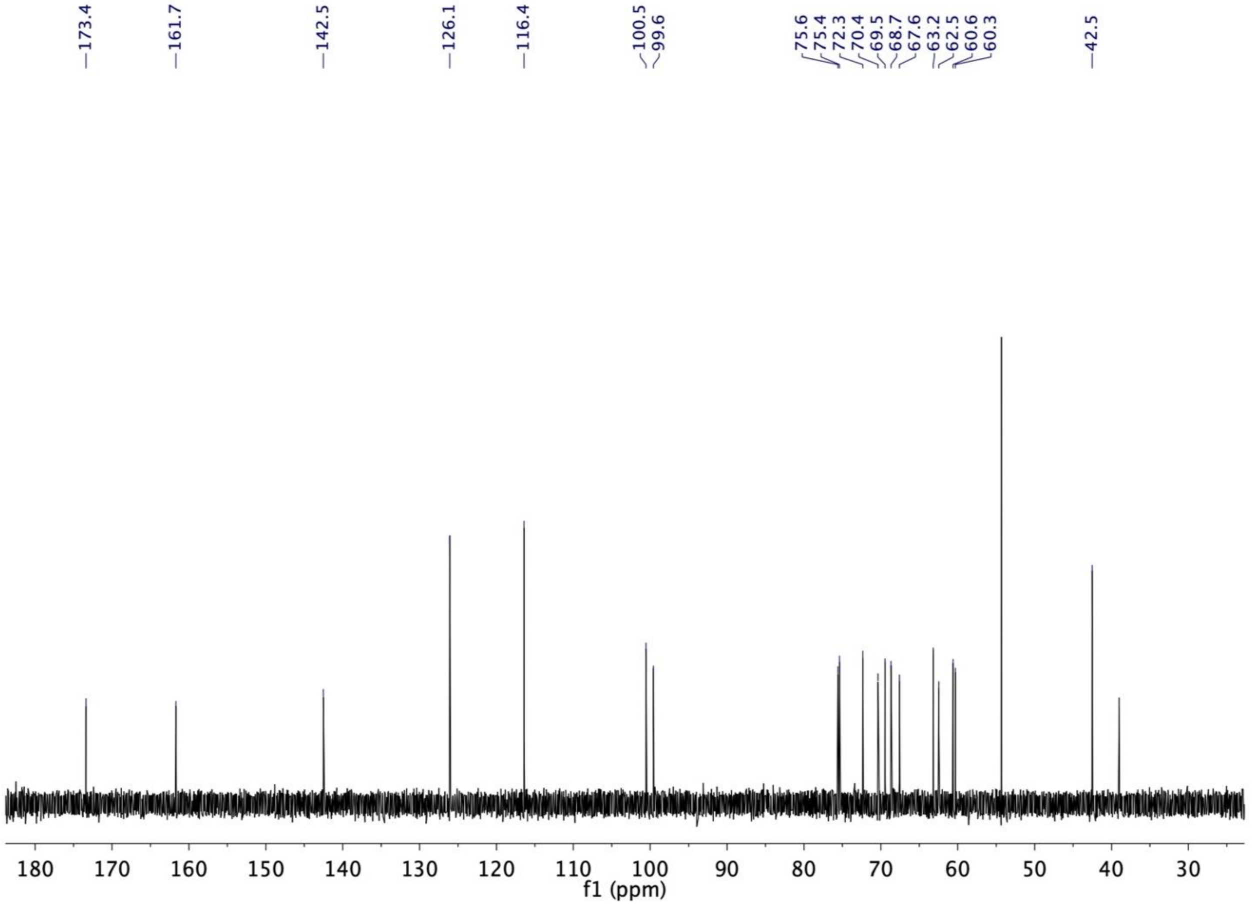

^{1}H and ^{13}C NMR spectra of Neu5,7,9triN$_3$α2–3Galβ*p*NP (**7**)

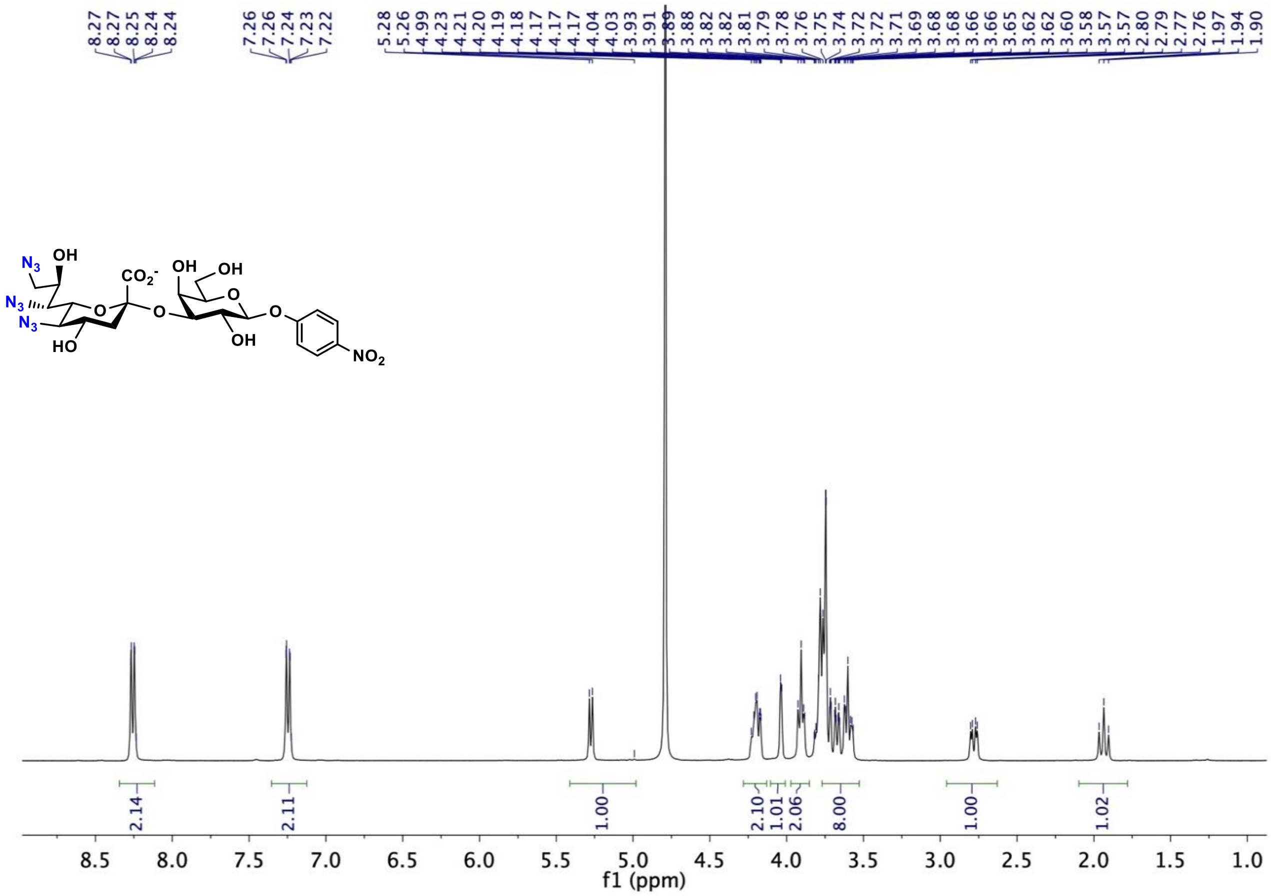

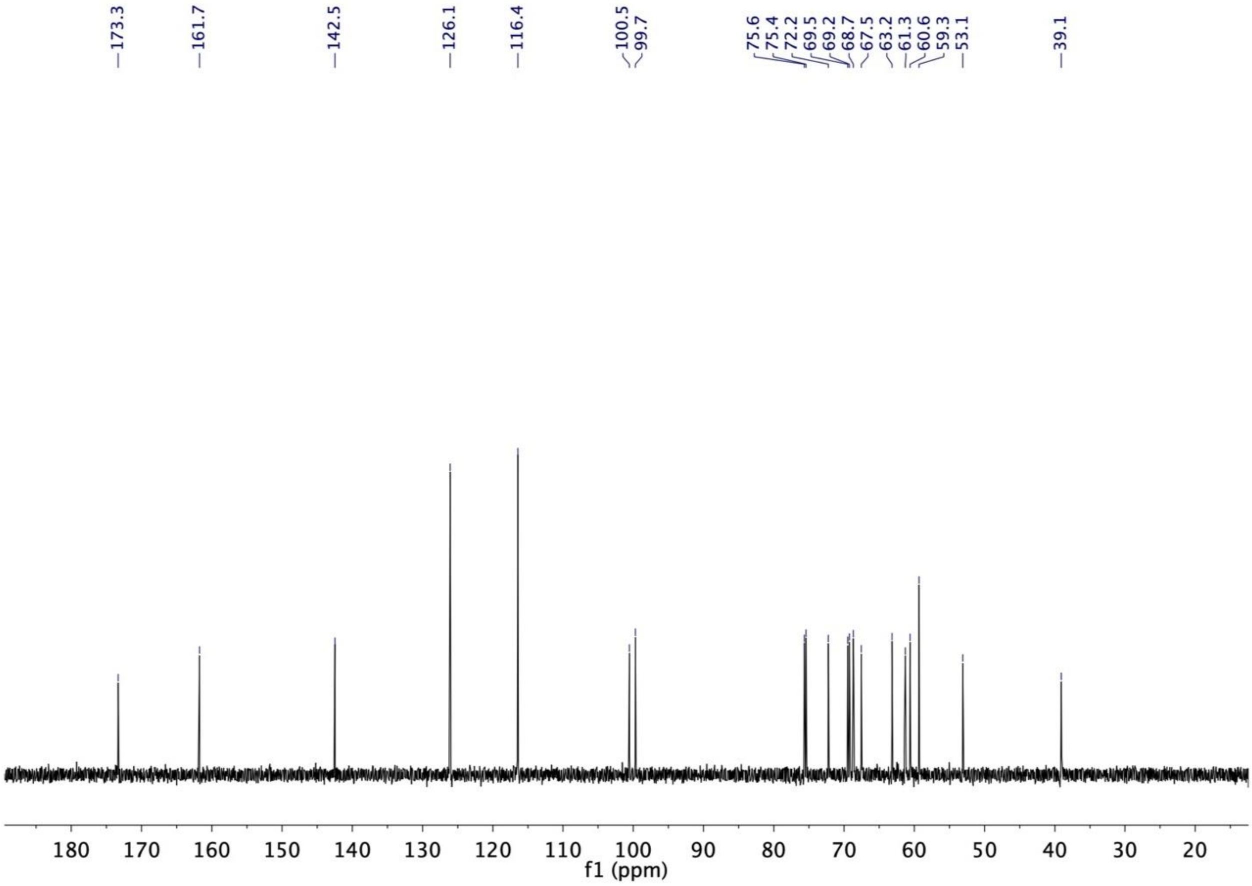

^{1}H and ^{13}C NMR spectra of Neu5,7diN$_3$α2–6Galβ*p*NP (**8**)

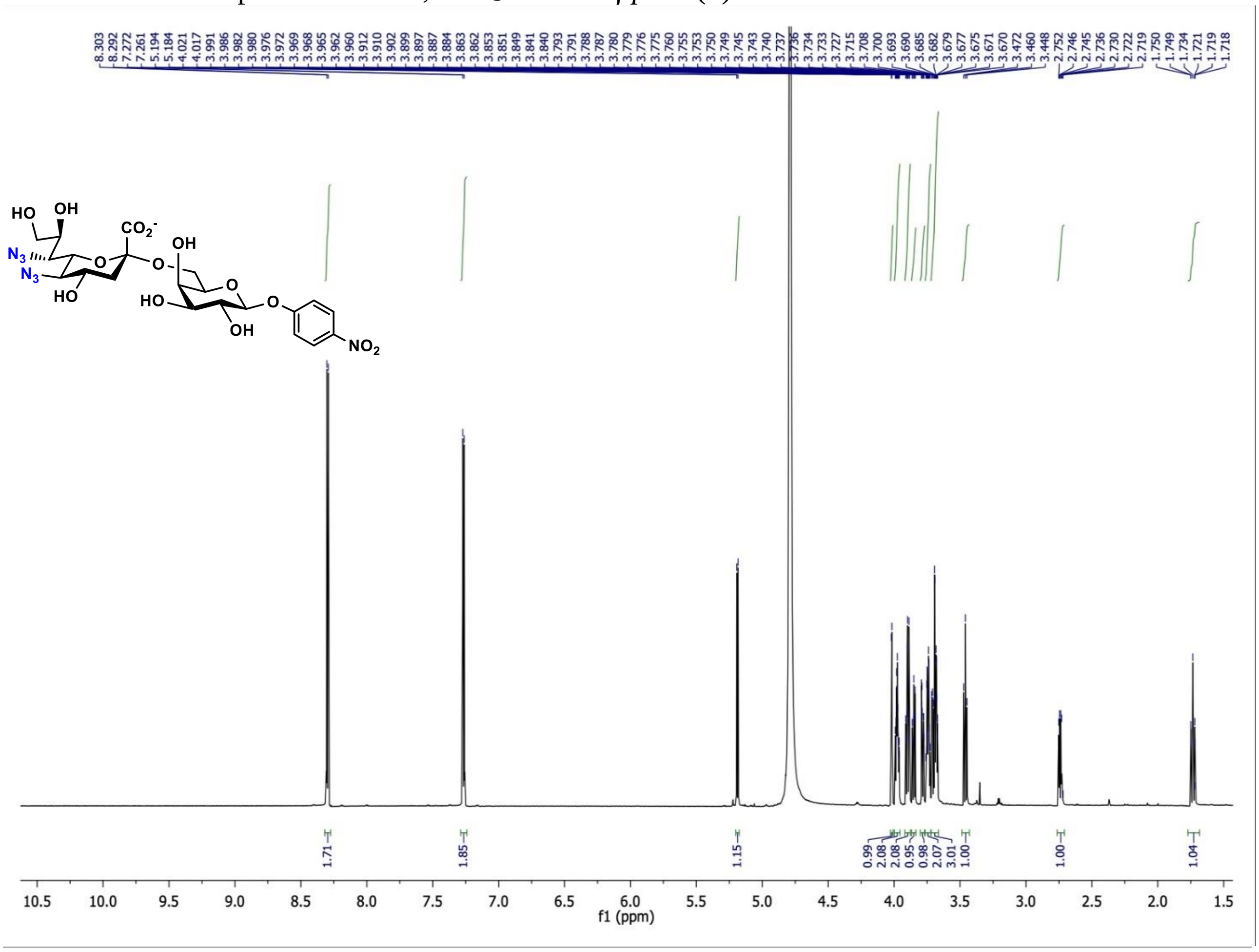

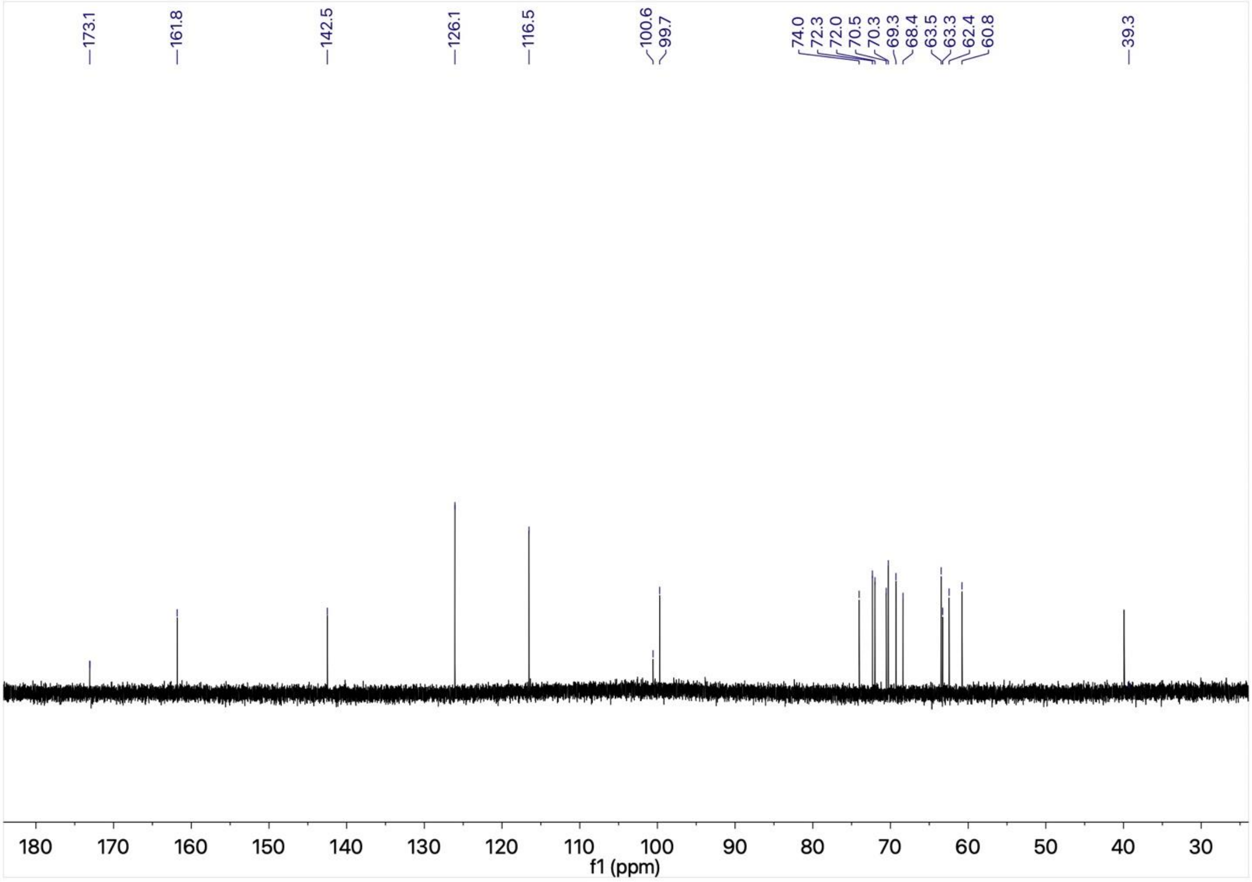

^{1}H and ^{13}C NMR spectra of Neu5,7,9triN$_3$α2–6Galβ*p*NP (**9**)

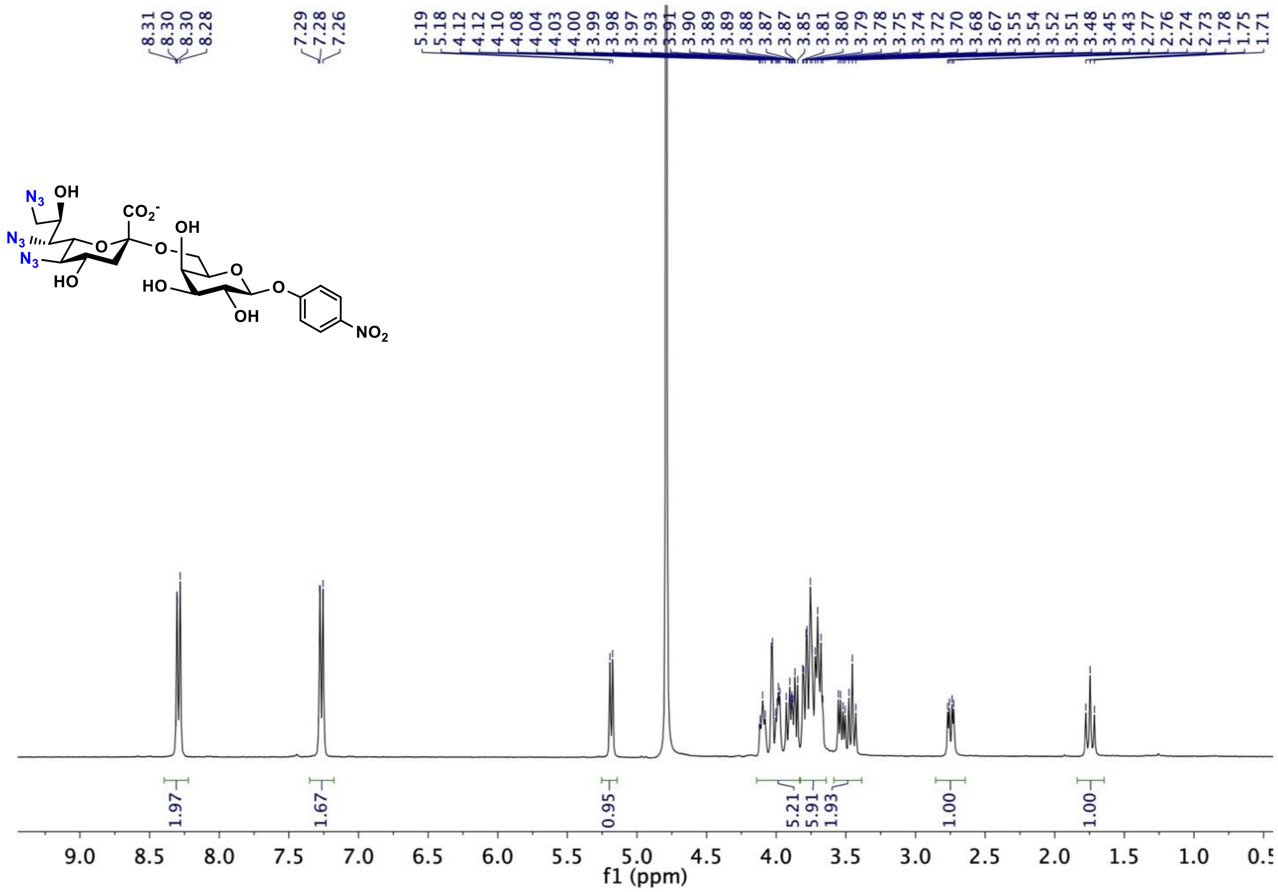

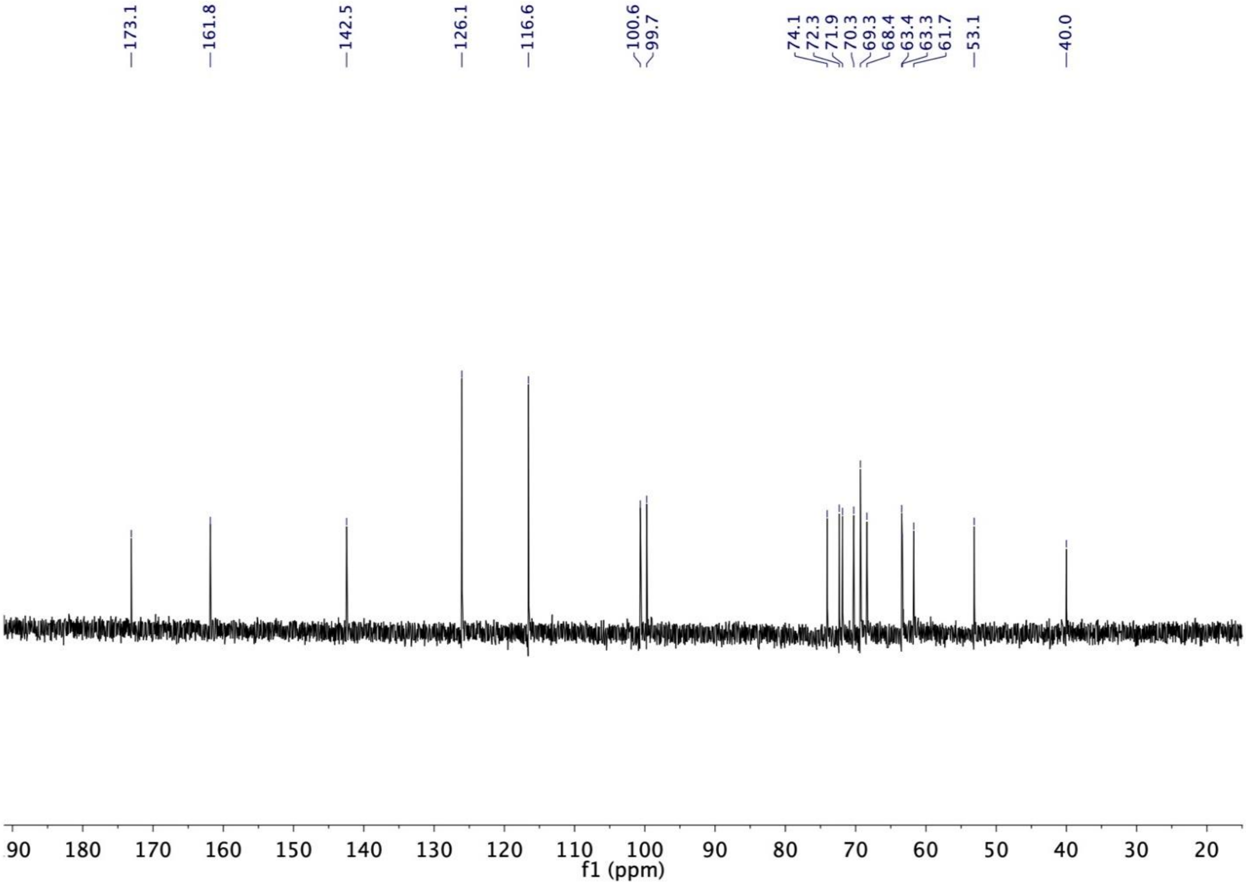

^{1}H and ^{13}C NMR spectra of Neu5Ac7NAcα2−3Galβ*p*NP (**10**)

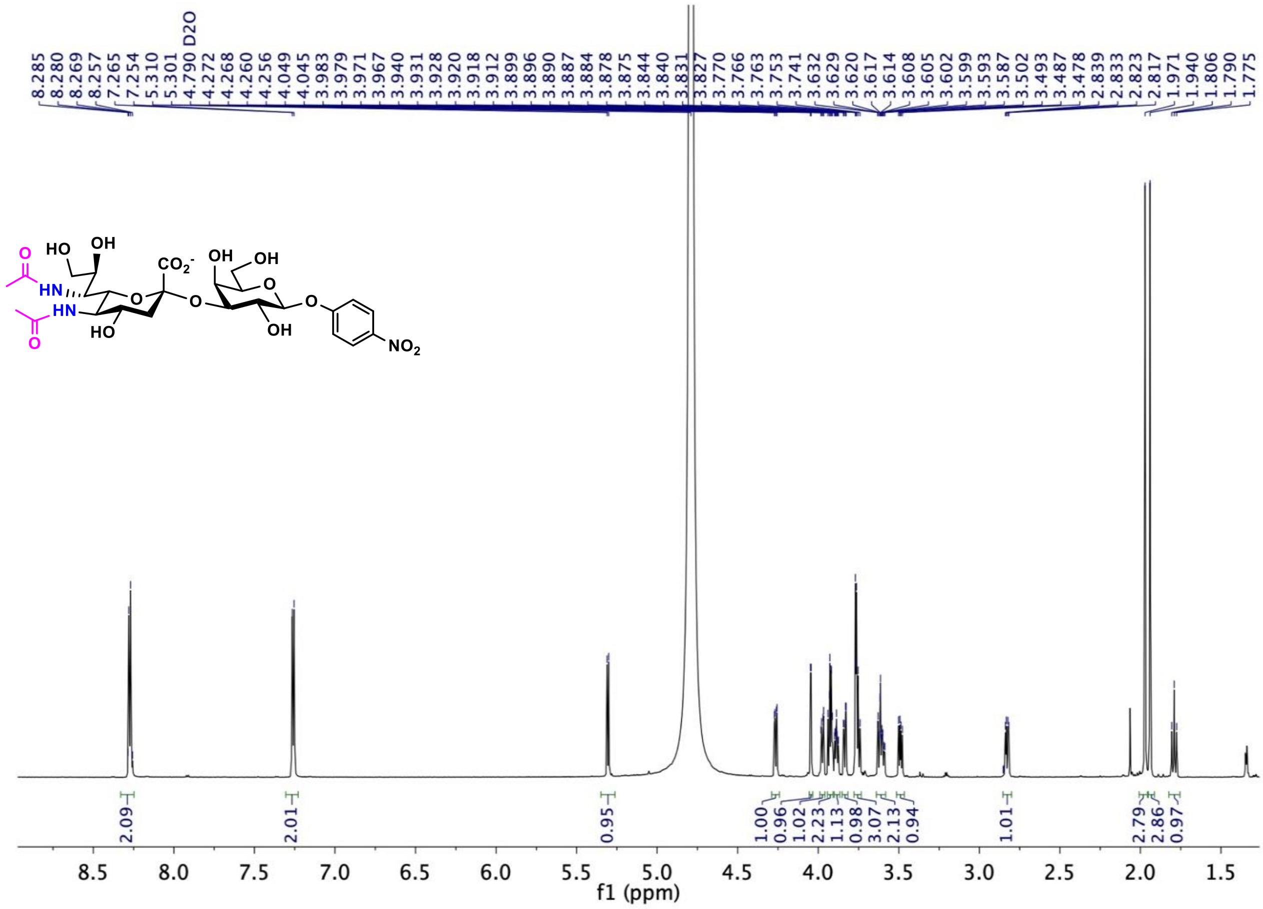

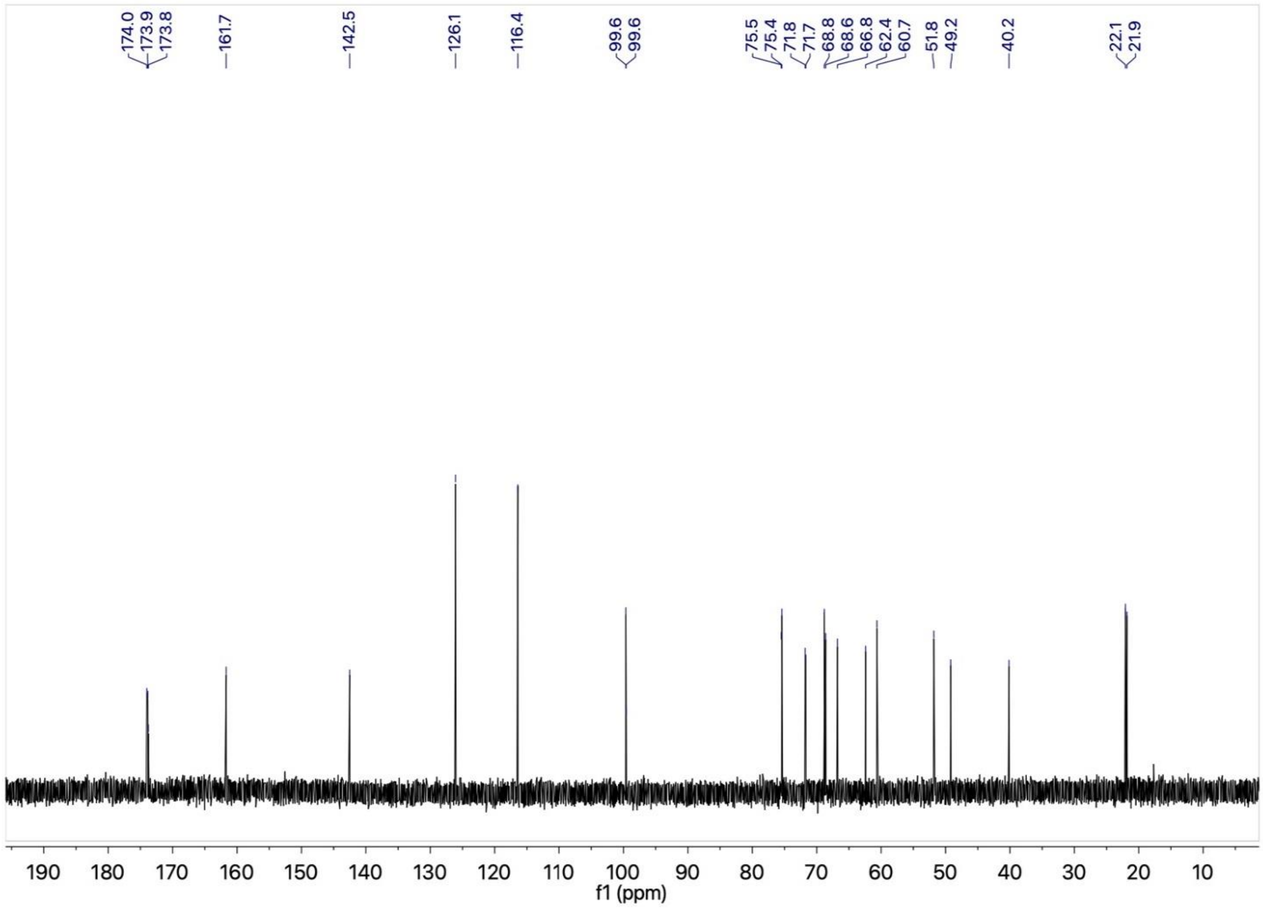

^{1}H and ^{13}C NMR spectra of Neu5Ac7,9diNAcα2−3Galβ*p*NP (**11**)

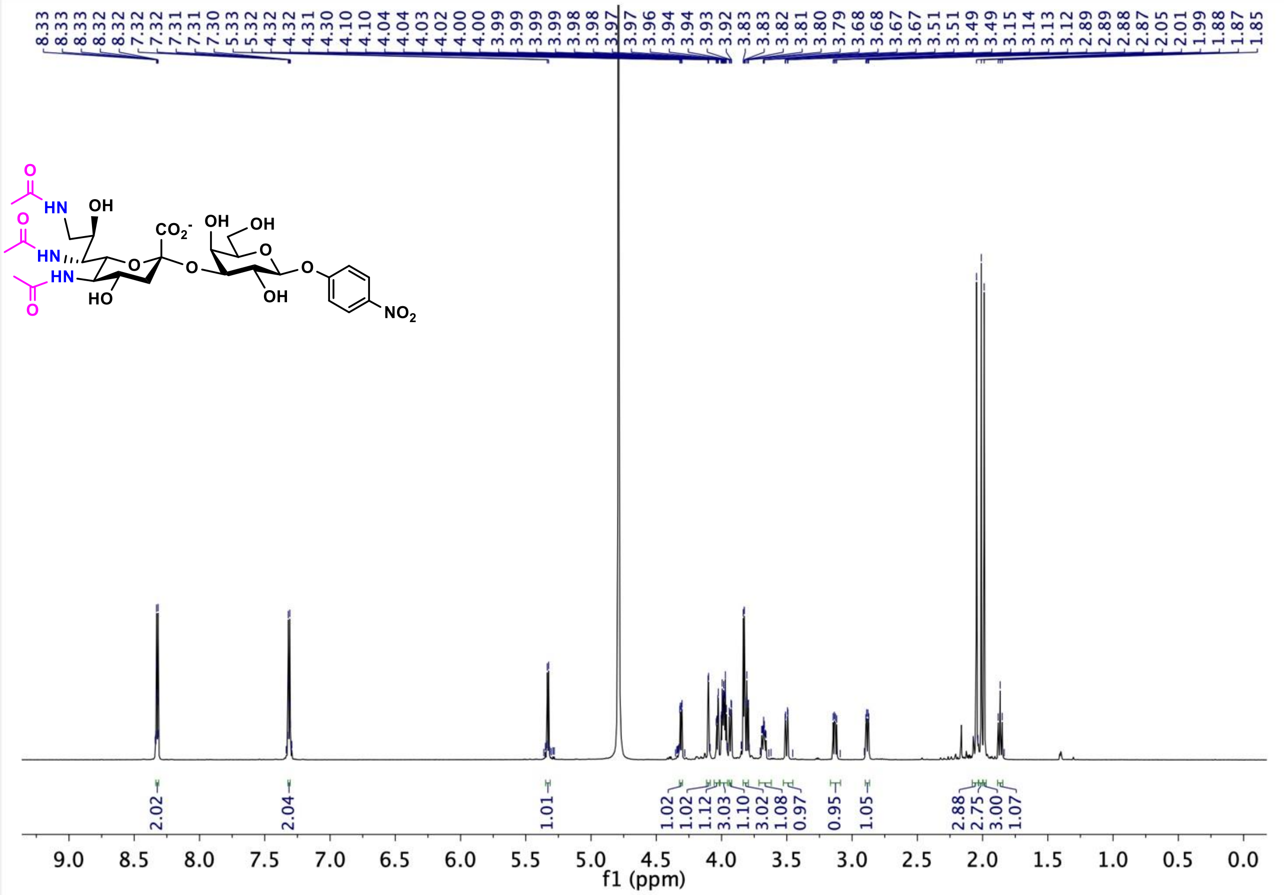

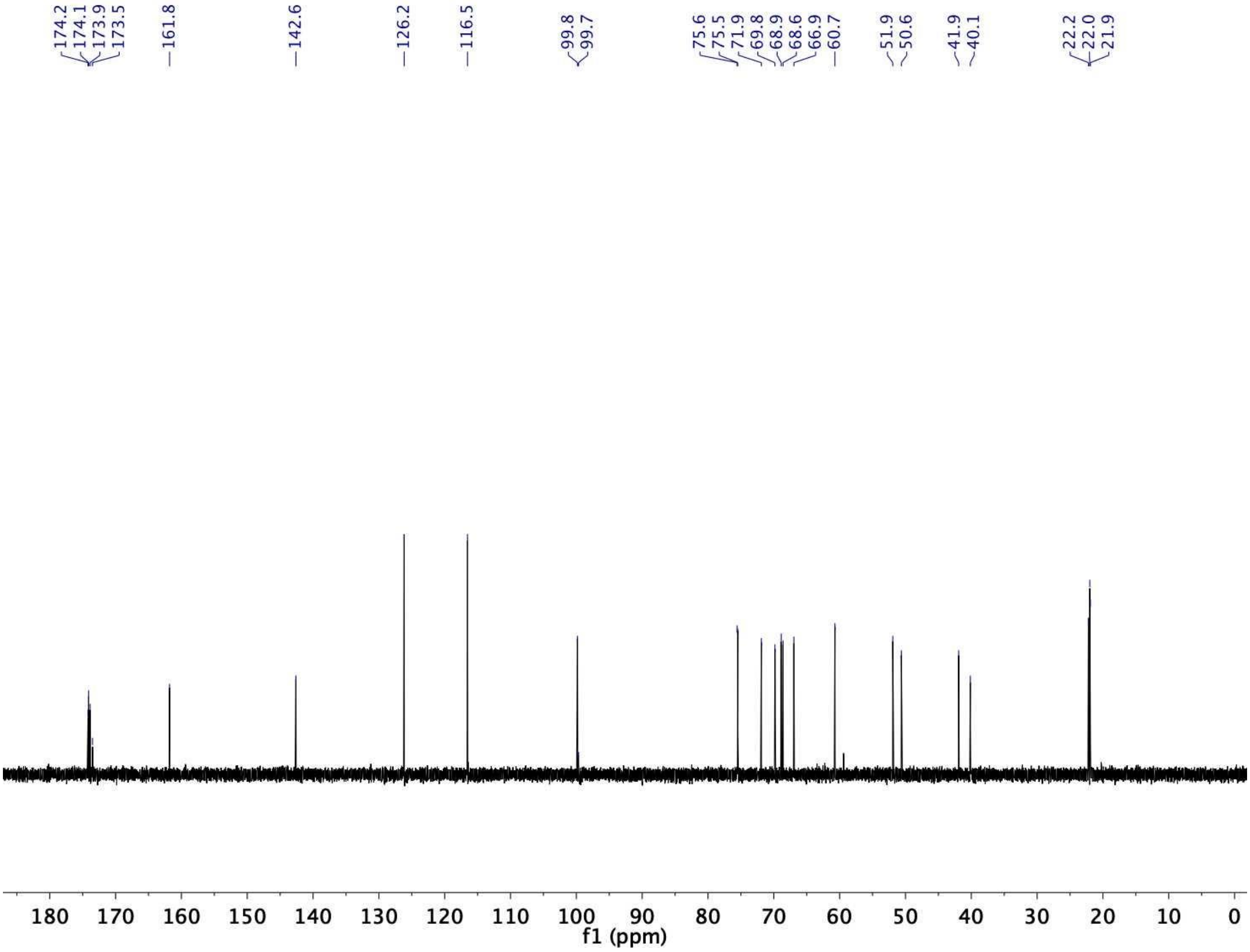

^{1}H and ^{13}C NMR spectra of Neu5Ac7NAcα2−6Galβ*p*NP (**12**)

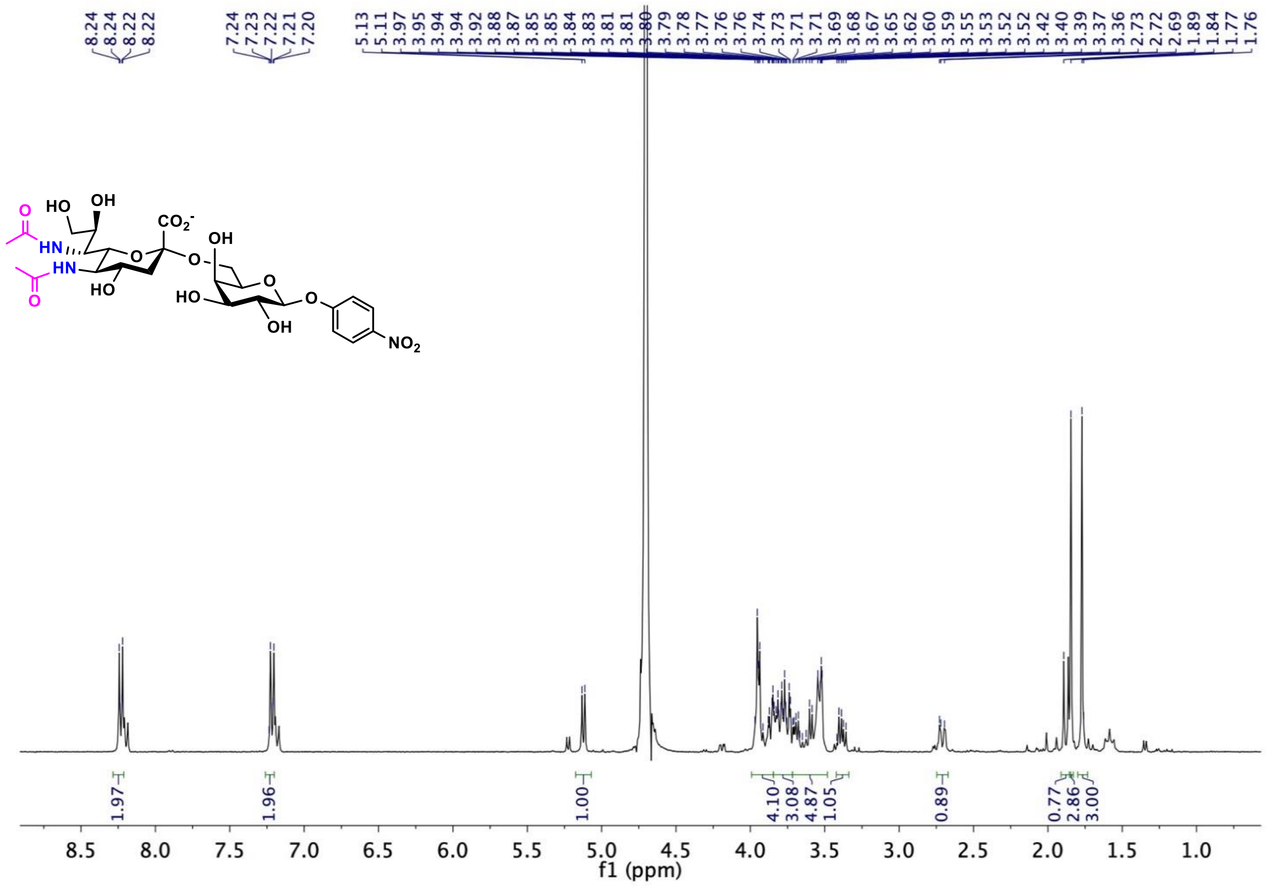

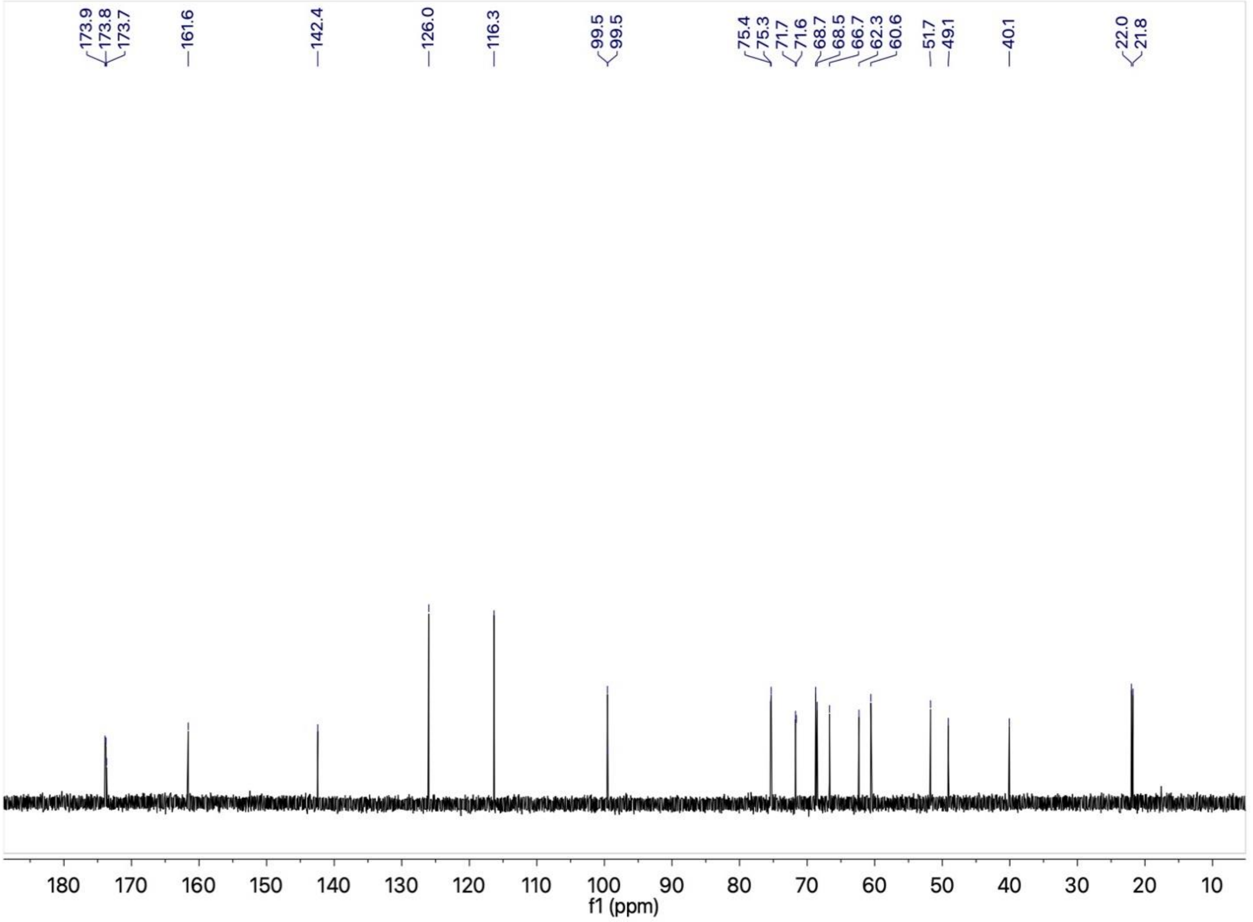

^{1}H and ^{13}C NMR spectra of Neu5Ac7,9diNAcα2−6Galβ*p*NP (13)

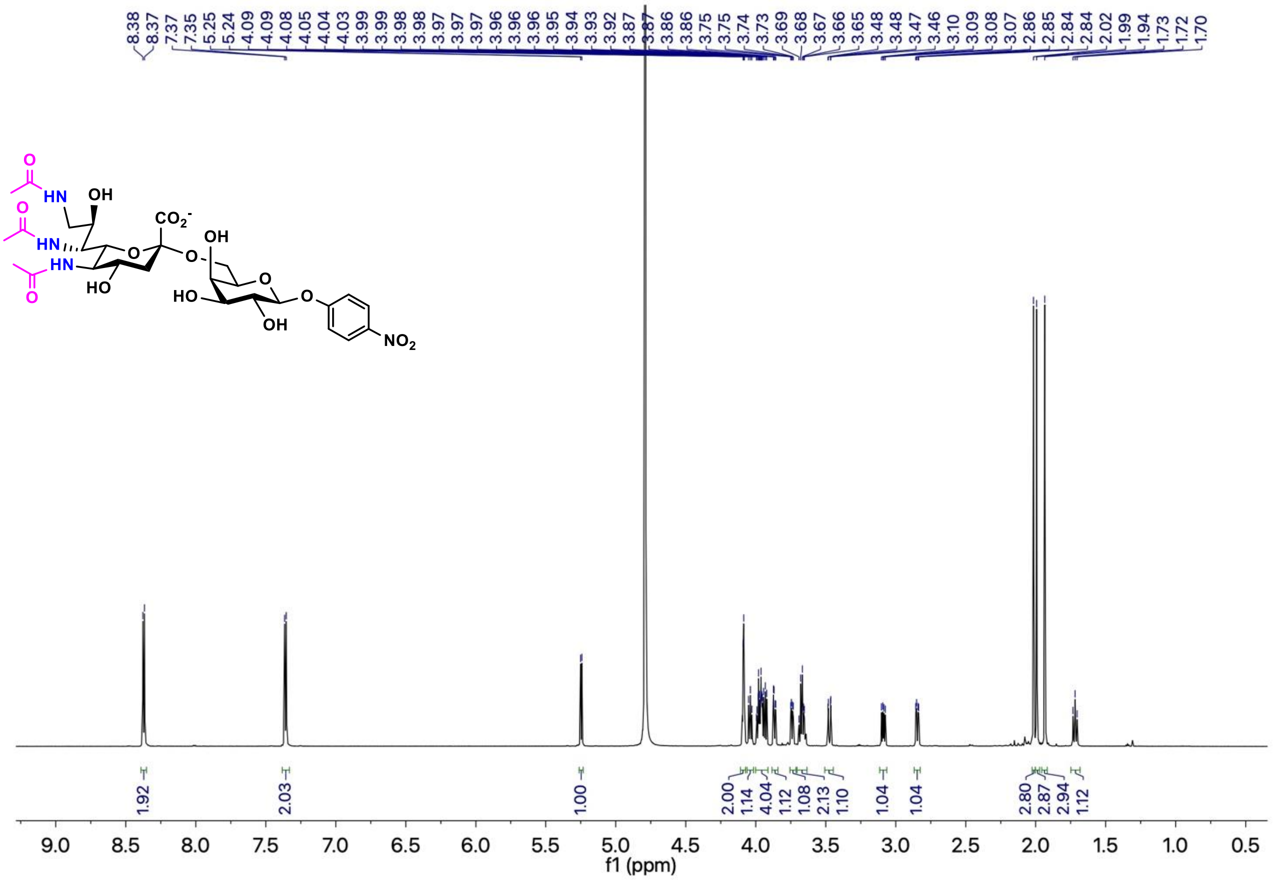

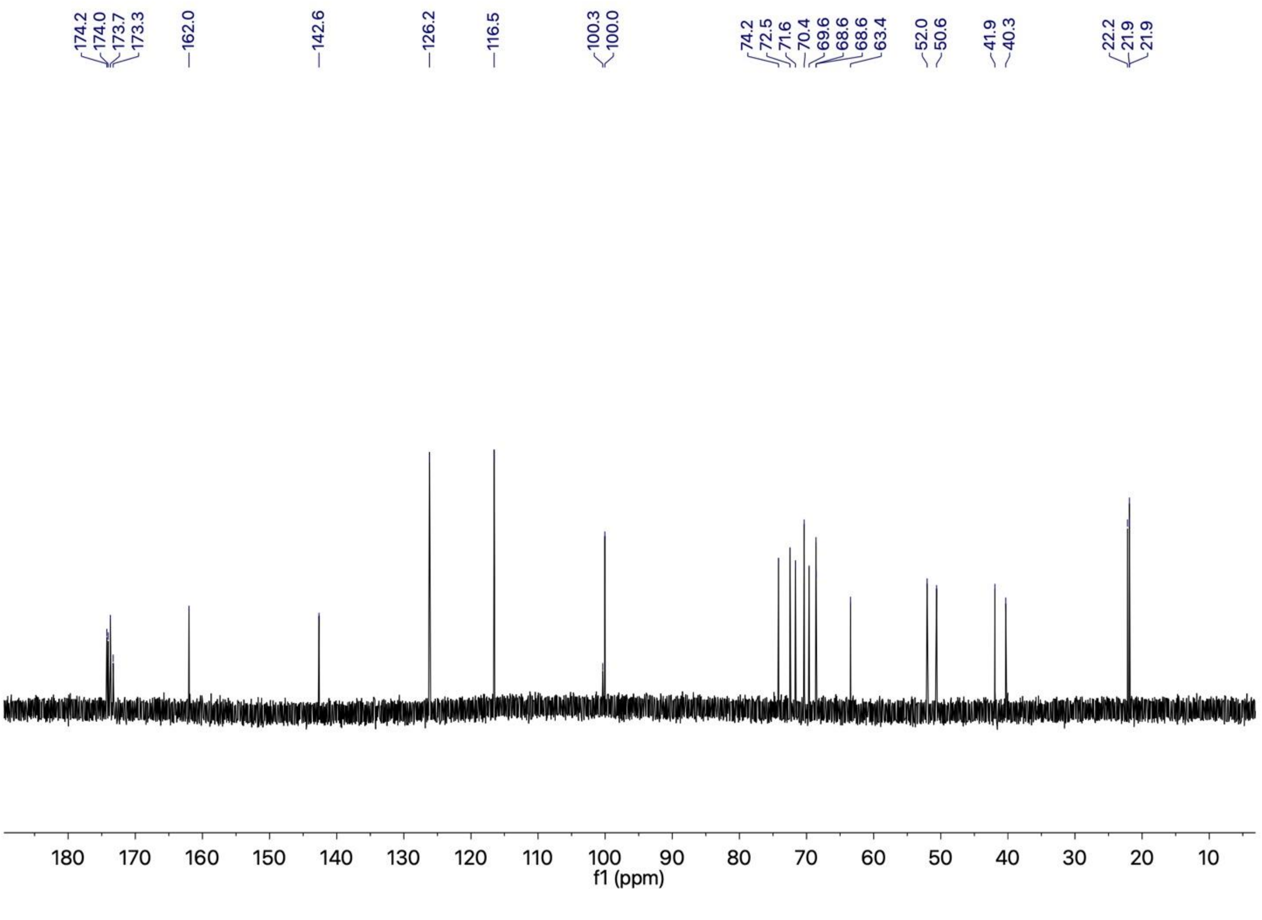

Supplement: Supplementary file 1 — id2c00502_si_001.pdf [file id2c00502_si_001.pdf]
